# PRMT5 regulates epigenetic changes in suppressive Th1-like iTregs in response to IL-12 treatment

**DOI:** 10.3389/fimmu.2023.1292049

**Published:** 2024-01-08

**Authors:** Nidhi Jadon, Sudarvili Shanthalingam, Gregory N. Tew, Lisa M. Minter

**Affiliations:** ^1^ Graduate Program in Animal Biotechnology and Biomedical Sciences, Department of Veterinary and Animal Sciences, University of Massachusetts Amherst, Amherst, MA, United States; ^2^ Department of Veterinary and Animal Sciences, University of Massachusetts Amherst, Amherst, MA, United States; ^3^ Department of Polymer Science & Engineering, University of Massachusetts Amherst, Amherst, MA, United States

**Keywords:** aplastic anemia (AA), bone marrow failure (BMF), protein arginine methyltransferase 5 (PRMT5), T helper type 1-like induced regulatory T cells (Th1-like iTregs), interleukin-12 (IL-12), sirtuin 1 (SIRT1), post-translational modifications (PTMs), symmetric histone di-methylation (H3R2me2s)

## Abstract

**Background:**

Induced regulatory T cells (iTregs) are a heterogeneous population of immunosuppressive T cells with therapeutic potential. Treg cells show a range of plasticity and can acquire T effector-like capacities, as is the case for T helper 1 (Th1)-like iTregs. Thus, it is important to distinguish between functional plasticity and lineage instability. Aplastic anemia (AA) is an autoimmune disorder characterized by immune-mediated destruction of hematopoietic stem and progenitor cells in the bone marrow (BM). Th1-like 1 iTregs can be potent suppressors of aberrant Th1-mediated immune responses such as those that drive AA disease progression. Here we investigated the function of the epigenetic enzyme, protein arginine methyltransferase 5 (PRMT5), its regulation of the iTreg-destabilizing deacetylase, sirtuin 1 (Sirt1) in suppressive Th1-like iTregs, and the potential for administering Th1-like iTregs as a cell-based therapy for AA.

**Methods:**

We generated Th1-like iTregs by culturing iTregs with IL-12, then assessed their suppressive capacity, expression of iTreg suppression markers, and enzymatic activity of PRMT5 using histone symmetric arginine di-methylation (H3R2me2s) as a read out. We used ChIP sequencing on Th1 cells, iTregs, and Th1-like iTregs to identify H3R2me2s-bound genes unique to Th1-like iTregs, then validated targets using CHiP-qPCR. We knocked down PRMT5 to validate its contribution to Th1-like iTreg lineage commitment. Finally we tested the therapeutic potential of Th1-like iTregs using a Th1-mediated mouse model of AA.

**Results:**

Exposing iTregs to the Th1 cytokine, interleukin-12 (IL-12), during early events of differentiation conveyed increased suppressive function. We observed increased PRMT5 enzymatic activity, as measured by H3R2me2s, in Th1-like iTregs, which was downregulated in iTregs. Using ChIP-sequencing we discovered that H3R2me2s is abundantly bound to the *Sirt1* promoter region in Th1-like iTregs to negatively regulate its expression. Furthermore, administering Th1-like iTregs to AA mice provided a survival benefit.

**Conclusions:**

Knocking down PRMT5 in Th1-like iTregs concomitantly reduced their suppressive capacity, supporting the notion that PRMT5 is important for the superior suppressive capacity and stability of Th1-like iTregs. Conclusively, therapeutic administration of Th1-like iTregs in a mouse model of AA significantly extended their survival and they may have therapeutic potential.

## 1 Introduction

After activation, naïve CD4 T cells can differentiate into a variety of T helper (Th) effector cell subsets (Th1, Th2, Th17, and follicular T helper cells) or into regulatory T cells (Tregs). Differentiation into these subsets is shaped by the type of antigen presented by antigen-presenting cells (APCs) and the surrounding cytokine milieu (1–3). Th1 cells can acquire immunopathological properties if directed against self-antigens. Thymically derived, naturally occurring CD4+CD25+ regulatory Tregs (nTregs) express the transcription factor Foxp3 and control the activation and expansion of T cells to provide immune homeostasis and control autoimmunity (4–6). Tregs constitute an attractive therapeutic tool for autoimmune disorders. Adaptive or induced Tregs (iTregs) can be expanded for therapeutic purposes from non-Treg precursors (naïve T cells) when stimulated in defined culture conditions supplemented with cytokines such as IL-2, TGFβ, and all-trans retinoic acid (4). Recent studies show that CD4+Foxp3+ iTregs can produce the pro-inflammatory cytokine IFNγ when stimulated in a Th1 cytokine environment, and polarizing T cells under these conditions makes iTregs sensors for inflammatory cytokines. These IFNγ-producing iTregs, or Th1-like iTregs, develop rapidly during inflammation and are the first to suppress initial immune responses (1).

In 2005, Sawitzki et al. demonstrated that immunizing mice with alloantigen, *in vivo*, generated IFNγ+ iTregs that protected mice from allograft rejection, while neutralizing IFNγ promoted skin graft necrosis (7). Volker et al., showed a correlation between the increased co-expression of IFNγ and Foxp3 in CD4+CD25+ peripheral blood lymphocytes (PBL) and improved graft function. In renal transplant patients, high numbers of CD3+CD4+CD25+IFNγ+ PBLs are associated with superior graft tolerance (8). Overall, IFNγ-producing Tregs have shown promising results in preventing graft rejection.

Treg-specific epigenetic patterns govern target gene transcription and translation and give clues as to Treg plasticity. Post-translational modifications (PTMs), including arginine methylation, regulate various biological processes such as gene transcription, cell cycle progression, and signal transduction. Protein arginine methyltransferases (PRMTs) are a group of enzymes that catalyze arginine (R) methylation on their target proteins. PRMTs are classified into 3 types depending on the type of methyl groups added to the R residues. Type I PRMTs (PRMT1, PRMT3, PRMT4, and PRMT6) catalyze asymmetric di-methylation, type II PRMTs (PRMT5 and PRMT9) catalyze symmetric di-methylation, and type III enzymes (PRMT7) drive mono-methylation of R residues (9–11). PRMTs utilize S-adenosylmethionine (SAMe) as their methyl donor, transferring a methyl group to terminal guanidine nitrogen atoms of R side chains on histones and other protein substrates (9, 12, 13).

Recent studies show R methyltransferase activity is upregulated in activated T cells (14, 15). PRMT5 is an epigenetic enzyme which has an essential function in biological processes such as RNA processing, DNA repair, and transcriptional regulation (16). PRMT5 is a major methyltransferase and catalyzes symmetric di-methylation (SDM) at the terminal amine group of arginine (17). PRMT5 deletion in T cells disrupts memory and effector T cell homeostasis and activation induced expansion, affecting T cell survival and cytokine signaling (18). PRMT5-deficiency in Tregs results in defective Treg maintenance and function, leading to a lethal scurfy-like autoimmune phenotype (19). While PRMT5 function in cell development, cell cycle progression, and T cell survival have been studied, its significance in Th1-like iTregs is still unclear.

Sirtuins (Sirt) are NAD^+^-dependent class III histone/protein deacetylase (HDAC) enzymes that are highly conserved among eukaryotes (20). Sirt1 is one of the seven mammalian homologues of the yeast transcriptional repressor, silent information regulator 2 (sir2). Sirt1 has many non-histone targets, including the transcription factors NF-κB, p53, and Foxo proteins (21). In a study performed by Beier et al., deleting *Sirt1* in CD4 T cells promoted Foxp3 expression and increased iTreg suppressive function, both *in vitro* and *in vivo.* Consistent with these data, deleting *Sirt1* in CD4 T cells prolonged the survival of major histocompatibility complex (MHC)-mismatched cardiac allograft (22). In a subsequent study, Akimova et al., demonstrated that the Sirt1 inhibitor, EX-527, ameliorates dextran sodium sulphate (DSS) colitis in immunocompromised mice (23). Kwon et al., have identified three novel acetylation sites on murine Foxp3 which are deacetylated by Sirt1 (K31, K262, and K267), and deacetylating Foxp3 leads to its proteasomal degradation (24). Therefore, downregulating Sirt1 generates stable and suppressive iTregs. However, it’s not known whether or how PRMT5 may act on Sirt1 in Th1-like iTregs to regulate their function.

In the developing field of personalized medicine, cell-based therapies for immunological disorders can minimize side effects and provide long-term management (25). Severe Aplastic Anemia (sAA) is an acquired bone marrow (BM) failure syndrome that is characterized by pancytopenia and BM hypoplasia. In most cases, disease etiology is unknown. Evidence shows that AA results from the active destruction of hematopoietic stem and progenitor cells by T lymphocytes, specifically by Th1 cells (26, 27). The first line of treatment for AA consists of hematopoietic stem cell transplant (HSCT). Immunosuppressive therapies (IST) are used when a matched sibling donor is not available, although both treatment options provide similar survival rates. For nearly 70% of individuals with sAA, a matching sibling donor is unavailable. As a result, IST becomes the treatment option for these patients. The current IST regimen typically involves the use of drugs such as anti-thymocyte globulin (ATG) and cyclosporine A (CsA). In a study performed by Shah et al. the long-term survival rate with IST approached approximately 70% for AA patients. However, the response rates varied over time. At 3 months of treatment, the overall response rate ranged from 41.8%, indicating improvement in blood counts and reduction in transfusion requirements, to 68.1% at 24 months. It’s important to note that only around 27.5% of patients achieve a complete response following 24 months of treatment (28). Furthermore, adverse side effects from IST can persist. Some of the most frequently observed adverse effects, such as febrile neutropenia, gum hypertrophy, and hypertension, align with those reported by other studies on IST (29). Allergic reactions like serum sickness are also anticipated and have been reported in a significant proportion of ATG recipients (28, 30). Infections and pneumonia are identified as the leading causes of death among AA patients receiving IST drug therapy. Intracranial hemorrhage is another significant concern associated with AA patients undergoing IST (30). Adoptively transferring Tregs presents an appealing alternative treatment approach for patients with acquired AA when a suitable donor is unavailable to improve overall survival and reduce the side effects of available medication. This study specifically aims to investigate the potential of using Th1-like iTregs as a cell-based immunosuppressive therapy for AA, while also seeking to understand the mechanisms by which Th1-like iTregs exert their suppressive effects.

Here, we confirmed that culturing iTregs with IL-12 generates a specific population of Th1-like iTregs with increased suppressive function, and demonstrate for the first time, these Th1-like iTregs harbor specific PRMT5-mediated symmetric demethylation on histones (H3R2me2s). Furthermore, knocking down PRMT5 in Th1-like iTregs, using siRNA or cell-penetrating anti-PRMT5 antibody approaches, attenuated their superior suppressive function. We also used ChIP sequencing to explore H3R2me2s-mediated transcriptional regulation across the genome. We discovered PRMT5 negative regulates *Sirt1* through H3R2me2s-mediated transcriptional silencing. Therefore, we conclude that the IL-12-PRMT5 axis is important to generate Th1-like iTregs and confers their stable and suppressive phenotype. Finally, using a mouse model of AA, we show that administering Th1-like iTregs under clinically relevant conditions provides a significant survival benefit, underscoring the potential of this cell-based therapy as a treatment for this Th1-mediated autoimmune BMF disease.

## 2 Materials and methods

### 2.1 Animals

All animal studies were approved by and conducted under the oversight of the Institutional Animal Care and Use Committee of the University of Massachusetts Amherst. Seven-weeks-old female C57BL/6 mice and the female F1 progeny of C57BL/6 x Balb/c crosses were obtained from the Jackson Laboratory (Bar Harbor, ME, USA). Mice were rested for 1 week, then were used for experiments. Age and sex-matched mice between 7-12 weeks were used in the study.

### 2.2 Aplastic anemia induction, scoring, and treatment

C57BL/6 x Balb/c F1 mice were irradiated with 2.5 Gy using a ^137^Cs source. Approximately 4 hours after irradiation, AA was induced by intraperitoneally (IP) injecting 5 x 10^7^ splenocytes obtained from age- and sex-matched C57BL/6 donors. The scoring criteria are based on body weight, posture, fur texture, skin, and activity. The scoring baseline is set from 0-2, with 2 being the highest score for any single criteria. Mice were treated with 2.5 million iTregs, injected through the retro-orbital sinuses, on day 12 and day 16 post-AA induction. For endpoint studies, mice were humanely euthanized on day 17 for further analysis. For mice on survival studies, mice were humanely euthanized when they could no longer eat or drink, lost 20% of their body weight due to disease progression, or reached a clinical score of “8” based on a standardized scoring rubric. Bone marrow cells were collected by flushing tibias and femurs of the legs using Hanks Balanced Salt Solution (HBBS; ThermoFisher Scientific, Waltham, MA). Spleens were passed through a 40 μM filter to obtain single-cell suspensions. Peripheral blood was obtained through cardiac puncture and red blood cells were lysed using ACK buffer, while white blood cells (WBCs) were counted using the Trypan Blue exclusion method. Data were acquired using a BD LSR Fortessa Flow Cytometer (BD Biosciences, San Jose, CA) to study CD4, CD8, Tbet, and Foxp3 expression and were analyzed using FlowJo software (BD Biosciences).

### 2.3 Antibodies and vital dyes

Antibodies used in this study: anti-hamster IgG, whole molecule (Sigma-Aldrich, St. Louis, MO), NA/LE hamster anti-mouse CD3ε, anti-mouse CD28 (BD Biosciences), anti-PRMT5 (A-11, Santa Cruz Biotechnology, Dallas, Tx), anti-H3R2me2s polyclonal (Invitrogen, Waltham, MA), alpha-tubulin mouse (DM1A, Cell Signaling Technologies (CST), Danvers, MA), anti-mouse secondary-IgG HRP linked F(ab’)_2_ fragment (clone NA9310V Amersham Biosciences, Piscataway, NJ), anti-rabbit secondary – IgG HRP linked whole antibody (clone NA934V, Amersham Biosciences), anti-PRMT5 rabbit monoclonal (clone ST51-06, Invitrogen), anti-H3R2me2s ChIP-seq grade (EpigenTek, Farmingdale, NY), anti-CD4 FITC (Clone H129.19, BD Biosciences), anti-CD25 (clone PC61, BioLegend, San Diego, CA), anti-Foxp3 (clone MF-14, BioLegend), anti-IFNγ (BD Biosciences), anti-Tbet (BioLegend), nuclear stain Draq5 (ThermoFisher Scientific), anti-rabbit IgG Fab_2_ Alexafluor 488 (CST), zombie violet fixable viability kit (BioLegend), CytoTell UltraGreen and CytoTell Red650 (AAT Bioquest, Pleasanton, CA).

### 2.4 *In vitro* iTreg, Th1, and Th1-like iTreg differentiation assays

CD4 T cells were isolated from spleens of C57BL/6 mice using the Mojosort™ CD4 T cell isolation kit (BioLegend) and resuspended in iTreg differentiation media supplemented with 10ng/ml IL-2 (BioLegend), 10ng/ml TGFβ (BioLegend), 80 ng/ml all-trans retinoic acid (Millipore Sigma, St. Louis, MO) and 2.5 μg/mL soluble anti-CD28 (BD Biosciences). Cells were seeded into wells of a 12-well tissue culture plate pre-coated with anti-hamster IgG (Sigma-Aldrich, St. Louis, MO) plus 1 mg/ml anti-CD3 (clone 145–2C11, BioLegend) and stimulated for 7 days at 37°C. Th1-like iTregs were generated by adding 10 ng/ml IL-12 (BioLegend) on day 3 only, or on days 3 and 5 of differentiation. The cells were collected on day 7 of differentiation. Th1 cells were generated by culturing CD4 T cells with plate-bound anti-CD3ϵ (5 μg/mL) plus anti-CD28 (2.5 μg/mL) in Th1 cell differentiation media supplemented with IL-2 (10ng/ml), IL-12 (10ng/ml), and anti-mouse IL-4 (1 *μ*g/ml; BioXcell, West Lebanon, NH). Th1 cells were collected for experiments on day 4 of differentiation. Media with a 1:1 mixture of RPMI 1640 and Dulbecco’s modified Eagle medium (GE Life Sciences, Pittsburgh, PA) supplemented with 10% FBS (Peak Serum, Wellington, CO), 2 mM L-glutamine, 1 mM sodium pyruvate, 100 U/mL penicillin, and 100 mg/mL streptomycin (GE Life Sciences) were used for cell culture.

### 2.5 Immunoblotting

Cells were lysed using RIPA digestion buffer, to prepare immunoblot samples. Proteins were separated by SDS-PAGE on 8%-10% gels. After transfer, the membranes were incubated for 1 hour in blocking solution (5% milk in Tris buffered saline with 1% Tween (TBST)). The membranes were washed using TBST and probed with primary antibody with 1% Bovine Serum Albumin (ThermoFisher Scientific) in TBST. The primary antibody was incubated overnight at 4°C, followed by incubating with the secondary antibody at room temperature the next day. The membranes were developed in Clarity Western ECL Substrate (Bio-Rad Laboratories, Hercules, CA). Antibodies used for immunoblotting: anti-PRMT5 (Santa Cruz), anti-H3R2me2s (Invitrogen), anti-alpha-tubulin (CST), anti-mouse secondary-IgG HRP linked F(ab’)_2_ fragment (clone NA9310V Amersham Biosciences), anti-rabbit secondary – IgG HRP linked whole antibody (clone NA934V, Amersham Biosciences).

### 2.6 Flow cytometry

Single-cell suspensions from *in vitro* generated iTregs or Th1-like iTregs were surface-stained with FITC-conjugated anti-CD4, APC-conjugated anti-CD25, BV421 zombie live-dead stain, PE-Cy7 conjugated anti-Tbet, and Alexa 700 anti-Foxp3. To quantify intracellular proteins, each sample was fixed and permeabilized according to the manufacturer’s directions using the Foxp3 staining buffer kit (Invitrogen). For intracellular IFNγ staining, cells were incubated with 50 ng/ml of PMA/ionomycin (Sigma Aldrich) in the presence of golgi plug (BD Biosciences) for 4 hours, then stained with PE conjugated anti-IFNγ. Data were acquired using a BD LSR Fortessa Flow Cytometer and analyzed using FlowJo software (BD Biosciences).

### 2.7 Protein subcellular localization using AMNIS imaging flow cytometry

Th1-like iTregs and iTregs were generated as described. Samples were fixed and permeabilized according to the manufacturer’s directions using the Foxp3 staining buffer kit and stained using anti-H3R2me2s (Invitrogen) followed by anti-rabbit IgG Fab2 Alexa Fluor (R) 488 (CST). Nuclei were stained using the cell permeable DRAQ5 (Thermo Scientific) fluorescent probe. Cells were visualized and quantified using an Image StreamX MK II imaging flow cytometer (Millipore Sigma). H3R2me2s nuclear localization was determined using the nuclear localization wizard and the IDEAS software to quantify proteins localized within the nucleus.

### 2.8 *In vitro* suppression assay

iTregs and Th1-like iTregs were differentiated as described. On day 7, iTregs or Th1-like iTregs (suppressors; Tsupp) were loaded with the cell tracker dye Red650 (APC fluorescence; AAT Bioquest). Splenocytes (responders; Tresp) were stimulated with soluble NA/LE hamster anti-mouse CD3ε + anti-mouse CD28 and crosslinked using hamster IgG, then stained with a different cell tracker dye, UltraGreen (FITC fluorescence; AAT Bioquest). To determine the iTreg to responder (Tsupp : Tresp) ratio for *in vivo* experiments in an AA mouse model, we seeded suppressor cells and responder cells at different ratios: 1:1, 1:10, and 1:20. For anti-IFNγ treatment, Th1-like iTregs and iTregs were treated with 1μg/ml of anti-IFNγ (BioXcell) on day 5 of polarization. These treated Th1-like iTregs were labeled with Red 650 and plated together with responder cells at the ratios described. Cells were co-cultured for 3 days, stained using zombie live-dead stain, and analyzed using flow cytometry. Percent suppression was calculated as follows: Suppression (%) = Area under the curve (AUC) of Responders without iTregs – AUC for Responders with iTregs/AUC for Responders without iTregs. AUC was calculated using ImageJ software (NIH.gov).

### 2.9 Mixed lymphocyte reaction and suppression assay

To generate bone marrow derived dendritic cells (BMDCs) from C57BL/6 x Balb/c F1 progeny, 106 BM cells/mL were cultured in RPMI 1640 medium (GE Life Sciences) supplemented with 10% FBS (Peak Serum), 2 mM L-glutamine, 100 U/mL penicillin, 100 mg/mL streptomycin (GE Life Sciences), and 20 ng/mL granulocyte macrophage colony-stimulating factor (BioLegend), and incubated at 37°C with 5% CO2. On days 2 and 4, half the media was removed and replaced with fresh media supplemented as above. On day 6, nonadherent cells were harvested and cultured in fresh supplemented media for 2 additional days. For mixed lymphocyte reaction, BMDCs were co-cultured at a ratio of 1:10 with bulk splenocytes from age- and sex-matched C57BL/6 mice, in a 1:1 mixture of RPMI 1640 and Dulbecco’s modified Eagle medium supplemented with 10% FBS, 2 mM L-glutamine, 1 mM sodium pyruvate, 100 U/mL penicillin, and 100 mg/mL streptomycin in 96-well round-bottom plates and incubated at 37°C with 7% CO2 for 12 days. For suppression assays, on day 7, iTregs or Th1-like iTregs (suppressors) were loaded with the cell tracker dye Red650. Splenocytes (responders) were stained with a different cell tracker dye, UltraGreen. Responder cells were seeded into 96-well U bottom plates, and suppressors were added to responders at the indicated ratios. Cells were co-cultured for 3 days, stained using zombie live-dead stain, and analyzed using flow cytometry. Suppression (%) = Area under the curve (AUC) of Responders without iTregs – AUC for Responders with iTregs/AUC for Responders without iTregs.

### 2.10 Chromatin immunoprecipitation sequencing and ChIP qPCR

Chromatin immunoprecipitation was performed using the EpiNext ChIP-Seq High-Sensitivity Kit (EpigenTek). Briefly, cells were crosslinked with 1% formaldehyde, lysed in Lysis buffer (EpiNext ChIP-Seq High-Sensitivity Kit), and sonicated in ChIP buffer (EpiNext ChIP-Seq High-Sensitivity Kit) with a Bioruptor Pico Sonicator System (Diagenode, Denville, NJ) using a 30- seconds-on – 30-seconds-off cycle for 30 minutes. Cell lysates with sheared chromatin were incubated overnight with ChIP sequencing grade anti-H3R2me2s (EpigenTek) bound to Dynabeads (Invitrogen), later DNA was purified using the manufacturer’s protocol, then sent for sequencing at LC Sciences, Houston, Texas. Purified DNA obtained using the manufacturer’s protocol was used as a template for ChIP qPCR.

### 2.11 siRNA transfection

siRNA transfection was performed 24 hours after plating CD4 T cells. 40 picomoles of control siRNA (Invitrogen), or PRMT5 siRNA (Invitrogen), were delivered using X-treme gene siRNA transfection reagent (Millipore Sigma) in optimum serum-free medium (Gibco). Cells were differentiated according to the standard differentiation protocol, described above and collected for analysis on day 7 for immunoblotting, qRT-PCR, and suppression assays.

### 2.12 Quantitative real time PCR

mRNA from differentiated cells was isolated using the quick RNA miniprep kit (Zymo Research, Irvine, CA). cDNA was prepared using recombinant rRNAsin (Promega Corporation, Madison, WI), MLV reverse transcriptase (Promega), dNTP (New England Biolabs, Ipswich, MA), and oligo(dt) (ThermoFisher Scientific). Cyber green (Bimake, Houston, TX) was used for qRT-PCR reactions. *PRMT5* TaqMan primers (Invitrogen) and *Actin* TaqMan primers (Invitrogen) with FAM dye were used to quantify *Prmt5* fold-expression. TaqMan Fast Advanced Master Mix (ThermoFisher Scientific) and RNase free water were used for TaqMAn qRT-PCR. The qRT-PCR reaction cycle was performed according to the manufacturer’s protocol.

### 2.13 Antibody delivery using cell-penetrating peptide mimics

CPPMs were generated in the laboratory of Dr. Gregory N. Tew (University of Massachusetts Amherst). 1 μM CPPM (P13D5) and 25 nM of anti-PRMT5 (Invitrogen) or IgG were complexed in PBS at a ratio of 40:1 for 30 minutes at 4°C with gentle rocking. CD4 T cells were isolated from C57BL/6 spleens using the Mojosort CD4 T cell isolation kit (BioLegend). Isolated CD4 T cells were treated with the CPPM-antibody complex for 4 hours at 37°C. Cells were harvested and washed twice with PBS. After washing, cells were plated and differentiated as described.

### 2.14 PRMT5 overexpression and retroviral transduction

pMRX- IRES-GFP retroviral vector, which contains a green fluorescent protein (GFP) reporter, was a kind gift from Dr. Leonid Pobezinsky (University of Massachusetts Amherst). The *PRMT5* construct was cloned into the pMRX- IRES-GFP vector by GeneScript company (Piscataway, NJ). Empty pMRX-IRES-GFP vector was used as a control. Retroviral supernatants were produced by transfecting Platinum-E (Plat-E) retroviral packaging cells (Cell Biolabs, Inc, San Diego, CA) using Transporter 5 transfection reagent (Polysciences, Warrington, PA). Retroviral supernatants were concentrated with PEG-it™ virus concentration reagent (System Biosciences, Palo Alto, CA) prior to transduction. CD4 T cells were retrovirally transduced 24 h after activation with 10x concentrated retrovirus supernatants by spin-infection (660 × *g* for 90 min at 37°C) in the presence of polybrene (4 μg/ml). Transduction media was replaced with Th1-like iTreg polarizing media 4 h after spin-infection. Transduced cells were analyzed by flow cytometry and qRT-PCR.

### 2.15 Plasmid extraction and purification

DH5α competent cells were transformed with *Prmt5* plasmids and amplified overnight in LB medium containing 10 *μ*g/ml carbenicillin using a 37°C shaker at 225 rpm. Bacteria were harvested and the *Prmt5* plasmid was extracted and purified using a plasmid extraction and purification kit (Takara, San Jose, CA) according to the manufacturer’s protocol.

### 2.16 Enzyme linked immunosorbent assay

CD4 T cells were isolated from spleens and polarized towards Th1-like iTregs and iTregs, as described. On day 7, cells were counted and re-stimulated using plate bound anti-CD3ϵ (5 μg/mL). After 24 hours of stimulation, the supernatants were collected for further analysis. For ELISAs, 96-well plates were pre-coated with anti-IFNγ or anti-IL-10 capture antibodies (BD Pharmingen) and incubated at 4°C overnight with gentle rocking. Next day, plates were washed and blocked in 1% BSA in PBS for 2 hours at room temperature. Supernatants were diluted with complete media, added to wells at desired concentrations and incubated at 4°C overnight with gentle rocking. Biotinylated anti-IFNγ or anti-IL-10 detection antibodies (BD Pharmingen) were added to wells and incubated for 1 hour at room temperature, followed by washing with PBST. Streptavidin HRP (horseradish peroxidase; BD Pharmingen) was added to wells and incubated for an additional hour, before adding TMB substrate (BD Biosciences). Samples were acquired using BioTek Synergy 2 plate reader (Winooski, Vermont), and analyzed using BioTek Gen5 software.

### 2.17 Flow cytometry multiplex immunoassay (LEGENDplex™)

We induced AA in C57BL/6 x Balb/c F1 mice, then administered therapeutic iTregs or Th1-like iTregs, as previously described. For endpoint studies, the mice were euthanized humanely on day 17 for further analysis. Peripheral blood was obtained by cardiac puncture, mixed with 100μl heparin (20 UI/ml) to prevent clotting, then centrifuged at 1000 x *g* for 10 minutes. Plasma cytokines were quantified using the LEGENDplex™ mouse inflammation panel (13-plex; BioLegend) following the manufacturer’s protocol. Results were analyzed using QOGNIT data analysis software (BioLegend).

### 2.18 Statistical analysis

All results are the mean ± SD and represent at least 3 independent experiments. Statistical analysis was performed using Prism 9 (Graphpad), unpaired two-tailed student’s *t*-test (comparison between two samples), one-way ANOVA (multiple comparisons used to study significance between samples using Dunnett’s multiple comparisons test), two-way ANOVA (multiple comparisons used for suppression assay using Šídák’s multiple comparisons test), multiple *t*-tests. Immunoblots were quantified using ImageJ software.

## 3 Results

### 3.1 Th1-like iTregs have increased suppressive capacity

**Figure 1:**
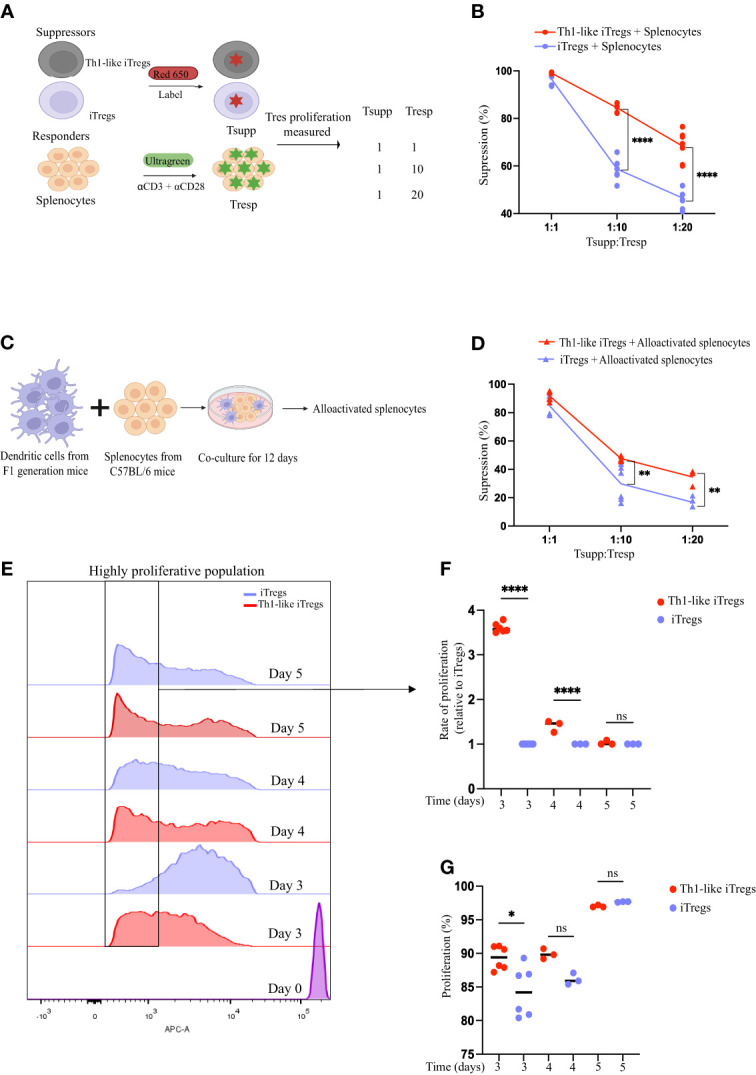
Th1-like iTregs have increased suppressive capacity. CD4 T cells were isolated from spleens of C57BL/6 mice, resuspended into iTreg differentiation media, and polarized for 7 days. Samples were left untreated or treated with IL-12 (10ng/mL) on day 3 only, day 5 only, or on days 3 and 5 both, of polarization. **(A)** On day 7, iTregs and Th1-like iTregs (Tsupp) were harvested and labeled with Red650. Splenocytes (Tresp) were harvested from C57BL/6 mice and labeled with UltraGreen dye to track proliferation. Tsupp and Tresp were co-cultured at concentrations of 1:1, 1:10, and 1:20 (Tsupp : Tresp). On day 3 of the co-culture, the percentages of proliferating responders were determined by flow cytometry, and the percent suppression was calculated. **(B)** Percent suppression quantified for Th1-like iTregs (n=8) and iTregs (n=8) co-cultured with responders that had been activated with soluble anti-CD3ϵ plus anti-CD28 then cross-linked with hamster IgG. **(C)** Experimental setup for mixed lymphocyte reaction (MLRs). Briefly, bone marrow derived dendritic cells (BMDCs) from C57BL/6 x Balb/c F1 progeny were co-cultured at a ratio of 1:10 with C57BL/6 splenocytes for 12 days. On day 12, MLRs were harvested, cells were labelled with UltraGreen dye, then used as responders in suppression assays. **(D)** Percent suppression quantified for Th1-like iTregs (n= 4) and iTregs (n=4) co-cultured with responders that had been alloactivated by cross-reacting dendritic cells in MLRs. **(E)** Representative histograms showing proliferating Th1-like iTregs and iTregs during the suppression assay. **(F)** Rate of proliferation of Th1-like iTregs and iTregs (relative to iTregs) collected from suppression assays on day 3 (n=6), day 4 (n=3), or day 5 (n=3) was calculated by gating on highly proliferative cells and analyzing the area under the curve (AUC) using ImageJ. **(G)** Percentage of proliferating Th1-like iTregs and iTregs collected from suppression assays on day 3 (n=6), day 4 (n=3), or day 5 (n=3). Data are the mean ± SD and are representative of at least 3 experiments. One-way ANOVA. *p < 0.05; ****p < 0.0001. Two-way ANOVA. **p < 0.01; ****p < 0.0001.

IFNγ-secreting Th1-like iTregs are generated when regulatory T cells (Tregs) encounter an alloantigen or Th1 cytokines such as IFNγ or IL-12 (1). Their generation is influenced by the microenvironment and immune signals present during antigen encounter or exposure to Th1 cytokines. We first set out to optimize a polarization protocol that generates Th1-like iTregs but also maintains Foxp3 expression to make iTregs sensors for inflammation. We isolated CD4 T cells from the spleens of C57BL/6 mice and added IL-12 into the iTreg differentiation media on day 3 and/or day 5 of a 7-day differentiation protocol. Using flow cytometry, we analyzed differentiated cells for Th1-like iTreg markers: CD4, CD25 (activation marker), Tbet (Th1 transcription factor), IFNγ (Th1 cytokine), and Foxp3 (iTreg transcription factor). We observed that adding IL-12 on day 3 of differentiation increased CD25, Foxp3, Tbet, and IFNγ expression, as measured by the mean fluorescence intensity (MFI), compared to iTregs that were treated on days 3 and 5 (**Supplementary Figures 1A–E**). Thus, for the remainder of our study we generated Th1-like iTregs by treating cells with IL-12 only on day 3 of differentiation. We evaluated Th1-like iTreg functional activity, using standard *in vitro* suppression assays using splenocytes as responder cells (Tresp). We stained the iTregs or Th1-like iTregs (Tsupp) with CytoTell Red650 dye while the Tresp were stained with CytoTell UltraGreen dye to track their proliferation, then mixed the cells at defined ratios of Tsupp : Tresp (**Figure 1A**). We chose these ratios because, in the spleen, the ratio of regulatory T cells to naïve T cells is approximately 1:10, while the therapeutic dose of iTregs we sought to evaluate in our AA mouse model is approximately 1:20 of the cell number used to induce disease. We collected cells from suppression assays after 3 days and analyzed proliferation using flow cytometry. Th1-like iTregs treated with IL-12 on day 3 of differentiation suppressed more potently at Tsupp : Tresp ratios of 1:10 and 1:20, compared to iTregs that were differentiated in the absence of IL-12 (**Figure 1B**). Not surprisingly, we did not observe differences in suppression between Th1-like iTregs and iTregs when these cells were mixed with responders at a 1:1 ratio, indicating that at high Tsupp : Tresp ratios, Th1-like iTregs and iTregs can suppress proliferating T cells equivalently. Rather, the superior suppressive capacity of Th1-like iTregs was observed when lower, more biologically relevant ratios of Tsupp : Tresp may be encountered. To understand how Th1-like iTregs respond to alloactivated splenocytes, we performed mixed lymphocyte reactions (MLR). We activated Tresp cells using allogenic bone marrow derived dendritic cells (BMDCs) from C57BL/6 x Balb/c F1 offspring, a co-culture system that mimics the *in vivo* mode of T cell activation in our mouse model of AA (**Figure 1C**). Impressively, Th1-like iTregs suppressed alloactivated splenocytes more efficiently than did iTregs (**Figure 1D**). These results confirm that Th1-like iTregs exhibit more robust suppressive activity, compared to iTregs not exposed to IL-12 early during the polarization process.

One explanation for the increased suppression mediated by Th1-like iTregs, especially at lower Tsupp : Tres ratios, might be that the Th1-like iTregs are more proliferative in culture than iTregs, effectively increasing the Tsupp : Tresp ratios in the 1:10 and 1:20 culture conditions. To address this, we examined proliferation of Th1-like iTregs and iTregs on days 3, 4, and 5 of the suppression assays. When we analyzed the highly proliferative populations on days 3 and 4, we observed that Th1-like iTregs had proliferated significantly more than the iTregs (**Figures 1E, F**). However, by day 5, these differences became statistically insignificant, when cells had likely reached their maximum proliferative capacity. Overall, Th1-like iTregs proliferated faster on day 3, compared to iTregs but, again, by days 4 and 5, this difference became non-significant as the rate of iTreg proliferation increased to match that of Th1-like iTregs (**Figure 1G**). Therefore, one explanation for why Th1-like iTregs are more potent suppressors in culture, may be due to their higher proliferation rates, compared to iTregs.

We also examined suppression markers, specifically CTLA-4 and PD-1, on Th1-like iTregs and iTregs. Th1-like iTregs exhibited more surface CTLA-4, while there was no significant difference observed for PD-1 expression (**Supplementary Figures 1F, G**, respectively). When we quantified cytokines secreted by regulatory T cells in suppression assays, we noted that Th1-like iTregs secreted more IL-10 and IFNγ than did iTregs (**Supplementary Figures 1H, I**, respectively). To further understand the impact of IFNγ on Th1-like iTregs during the polarization process, we treated Th1-like iTregs and iTregs with anti-IFNγ on day 5 of polarization. At the 1:10 ratio of Tsupp : Tresp, Th1-like iTregs maintained their potent suppressive activity compared to iTregs. However, they performed less well than did iTregs in their ability to suppress proliferating cells at the 1:20 Tsupp : Tresp ratio (**Supplementary Figure 1J**). We did not assess whether anti-IFNγ treatment slows Th1-like iTreg cell proliferation in suppression assays. Although we cannot rule this out, we conclude that any effects anti-IFNγ treatment had on Th1-like iTreg proliferation were subtle and not detected except at high Tsupp : Tresp ratios (1:20). Alternatively, inhibiting access to IFNγ during the polarization process may have impacted Th1-like suppressive capabilities in other ways (31). Collectively, our data show that compared to Tregs not exposed to IL-12 early during their polarization process, Th1-like iTregs exhibit superior suppressive activity and more robust proliferation in *in vitro* suppression assays, and these effects may in part be conveyed through IFNγ produced by Th1-like iTregs during their differentiation process.

### 3.2 IL-12 treatment upregulates PRMT5 activity in Th1-like iTregs

**Figure 2:**
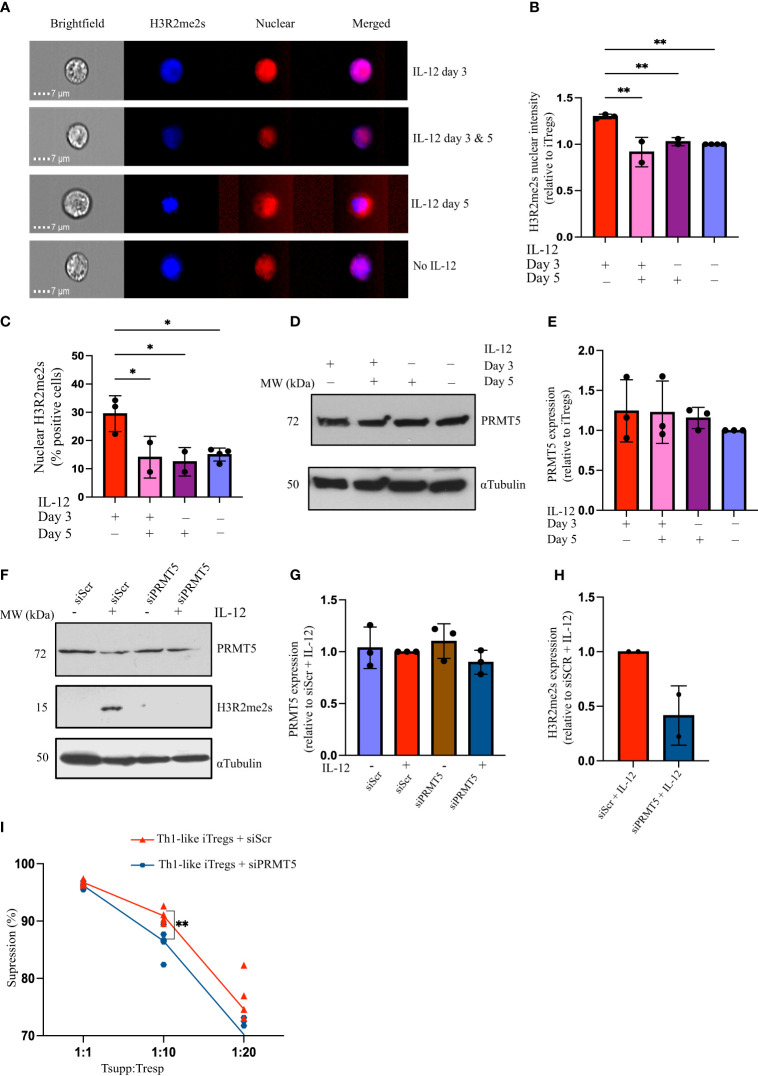
IL-12 treatment upregulates PRMT5 activity in Th1-like iTregs. **(A)** Representative images acquired using AMNIS imaging flow cytometry to analyze 2,000 iTregs, showing nuclear H3R2me2s in iTregs treated with IL-12 on day 3 and/or day 5 of differentiation, and **(B)** quantified H3R2me2s nuclear intensity (relative to iTregs). **(C)** Percent positive cells with H3R2me2s localized in the nucleus when treated with IL-12 on day 3 (n=3), on days 3 & 5 (n=2), on day 5 (n=2), or left untreated (n=4). **(D, E)** Immunoblot and quantification showing PRMT5 in iTregs treated with IL-12 on day 3 and/or day 5 of differentiation (n=3). CD4T cells were activated for 24 hours before siRNA transfection and differentiating towards iTregs as described. **(F)** PRMT5 and H3R2me2s expression and **(G, H)** quantification in iTregs transfected with scrambled (siScr) or PRMT5 siRNA (siPRMT5) and cultured without or with IL-12 on day 3 of polarization (n=3). **(I)** Percent suppression by Th1-like iTregs treated with siScr (n=6) or siPRMT5 (n=6), of responders activated with soluble anti-CD3ϵ plus anti-CD28 and cross-linked with hamster IgG. Data are the mean ± SD and are representative of at least 3 experiments. One-way ANOVA. *p < 0.05; **p < 0.01; multiple *t* tests. **p < 0.01.

Exposing CD4 T cells to IL-12 induces a specific differentiation pathway in iTregs, leading them to acquire Th1-like characteristics, produce IFNγ, and exhibit unique phenotypic and functional properties that distinguish them from conventional Tregs. Nagai et al. showed that knocking out PRMT5, specifically in Tregs, attenuates their suppressive capacity (19). However, whether PRMT5 modulates functional activity in Th1-like iTregs has not been thoroughly investigated. To assess how PRMT5 contributes to Th1-like iTreg differentiation, we studied the post-translational modifications (PTMs) it mediates. Symmetric arginine histone di-methylation (H3R2me2s) is specific to PRMT5; therefore, we used this as a marker for PRMT5 enzymatic activity. We observed more nuclear H3R2me2s in Th1-like iTregs treated with IL-12 on day 3 of differentiation, compared to iTregs treated with IL-12 on day 3 and/or 5, or left untreated (**Figures 2A, B**). The percentage of cells positive for nuclear H3R2me2s was also significantly greater for cells treated with IL-12 on day 3 of differentiation, compared to iTregs treated with IL-12 on day 3 and/or 5, or left untreated (**Figure 2C**). These results indicate there is likely a narrow window during which IL-12 exposure modulates PRMT5 function in Th1-like iTregs, and that transient (day 3 only), rather than continuous (day 3 and 5), exposure to IL-12 induces maximal PRMT5 activity. This may be temporal due to accessibility to chromatin, during which time symmetrically di-methylated histones bind to target DNA (i.e. during early brief exposure to IL-12 on day 3), while continuous exposure to IL-12 (i.e. day 3 and day 5 treatment) counteracts or supersedes this process, for instance by diminishing levels of Foxp3 and reducing IL-2 receptor expression necessary for iTreg differentiation (32). Additional in-depth studies are needed to provide additional mechanistic details.

We measured PRMT5 expression following IL-12 treatment and did not observe differences in PRMT5 expression, regardless of when iTregs were exposed to IL-12. This indicates that the increase in H3R2me2s seen after IL-12 treatment on day 3 can be attributed to differences in PRMT5 activity rather than changes in protein levels (**Figures 2D, E**). To confirm PRMT5 is necessary for the increased suppressive capacity of Th1-like iTregs, we knocked down *Prmt5* using siRNA. SiPRMT5 treatment reduced PRMT5 expression in Th1-like iTregs by day three (**Supplementary Figure 2A**). Although PRMT5 protein expression recovered by day 7 of differentiation, knocking down PRMT5 during the initial stages of differentiation abrogated H3R2me2s expression, even in Th1-like iTreg cells treated with IL-12 on day 3 of differentiation (**Figures 2F–H**). After siPRMT5 treatment, Th1-like iTregs exhibited higher levels of CD25 and Tbet, compared to control siRNA treatment, regardless of IL-12 treatment, but Foxp3 levels were not significantly different from controls (**Supplementary Figures 2B–D**). In response to IL-12 exposure, T-bet expression increased in Th1-like iTregs in which PRMT5 was knocked down, and IFNγ expression was higher in samples treated with IL-12, regardless of whether PRMT5 was knocked down (**Supplementary Figures 2D, E**, respectively). siPRMT5 treatment may disrupt lineage commitment towards suppressive Th1-like iTregs, as evidenced by increased CD25 and Tbet expression, while IFNγ expression seems less affected. We further noted that downregulating PRMT5 in Th1-like iTregs reduced their suppressive capacity compared to Th1-like iTregs treated with a control scrambled siRNA (**Figure 2I**), and this was also observed for iTregs (**Supplementary Figure 2F**). Collectively, these results indicate that functional PRMT5 is important for Th1-like iTregs, as well as iTregs, to maintain superior suppressive capacity, especially at higher Tsupp : Tresp ratios.

To confirm that PRMT5 activity is responsible for the increased suppression of Th1-like iTregs, we sought to inhibit PRMT5 using a second approach. To do this, we delivered an antibody specific for PRTM5 using a validated cell-penetrating peptide mimic (CPPM), a co-polymer comprised of blocks of 13 phenyl-containing and 5 diguanidine moieties (33). In previous studies, we successfully abrogated the enzymatic activity of Protein Kinase C theta (PKCθ) in human primary T cells and iTregs, using CPPM-phospho-PKCθ antibody delivery (33–35). Here, we complexed the CPPM with anti-PRMT5, or with anti-rabbit IgG as a control, then incubated CD4 T cells with the CPPM-antibody complex prior to polarizing towards Th1-like iTregs (**Supplementary Figure 3A**). We confirmed PRMT5 knockdown through immunoblot and quantified PRMT5 expression relative to control IgG delivery in Th1-like iTregs (**Supplementary Figures 3B, C**). When delivered into iTregs, CPPM:anti-PRMT5 increased CD25 and Foxp3 expression, and decreased Tbet expression, compared to delivering CPPM : IgG. However, treating Th1-like iTregs with CPPM:anti-PRMT5 did not significantly affect CD25, Foxp3, Tbet, or IFNγ expression, compared to delivering CPPM : IgG (**Supplementary Figures 3D–F**). Th1-like iTregs treated with CPPM:anti-PRMT5 did not increase IFNγ expression following IL-12 exposure, unlike those treated with CPPM : IgG (**Supplementary Figure 3G**), suggesting that CPPM:anti-PRMT5 delivery can disrupt IL-12 signaling pathways in Th1-like iTregs. We also performed *in vitro* suppression assays using Th1-like iTregs or iTregs treated with CPPM-anti-PRMT5. Supporting our results using siRNA approaches, PRMT5-knockdown reduced the suppressive activity of Th1-like iTregs and iTregs, both (**Supplementary Figures 3H, I**). These data support the notion that PRMT5-mediated symmetric di-methylation is an important contributor to Th1-like iTreg functional activity, but it also acts in iTregs to mediate suppression (19).

### 3.3 H3R2me2s differentially binds the *Sirt1* promoter in iTregs and Th1-like iTregs

**Figure 3:**
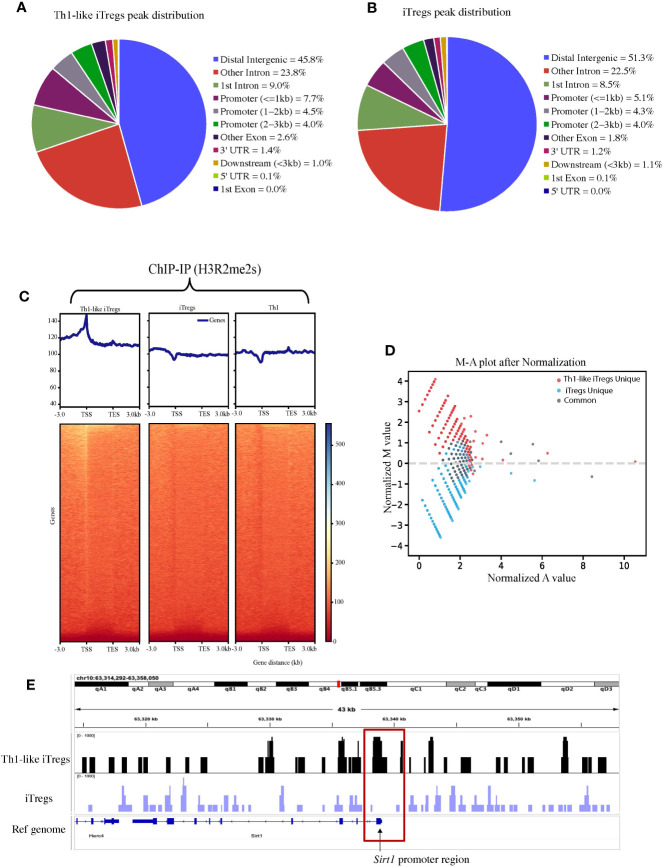
H3R2me2s differentially binds the *Sirt1* promoter in iTregs and Th1-like iTregs. We used ChIP sequencing to analyze the genes associated with H3R2me2s in Th1-like iTregs and iTregs. Peak distribution of genes H3R2me2s associates with in **(A)** Th1-like iTregs and **(B)** iTregs. **(C)** Representative graphs and heat maps of the genes identified by ChIP-IP sequencing that H3R2me2s associates with in Th1-like iTregs, iTregs, and Th1 cells. **(D)** We performed M-A norm analysis to identify Th1-like iTreg-unique (red), iTreg-unique (blue), and common (grey) genes that associate with H3R2me2s. **(E)** We visualized peaks at the *Sirt1* promoter using the integrative genomic viewer (IGV).

**Figure 4:**
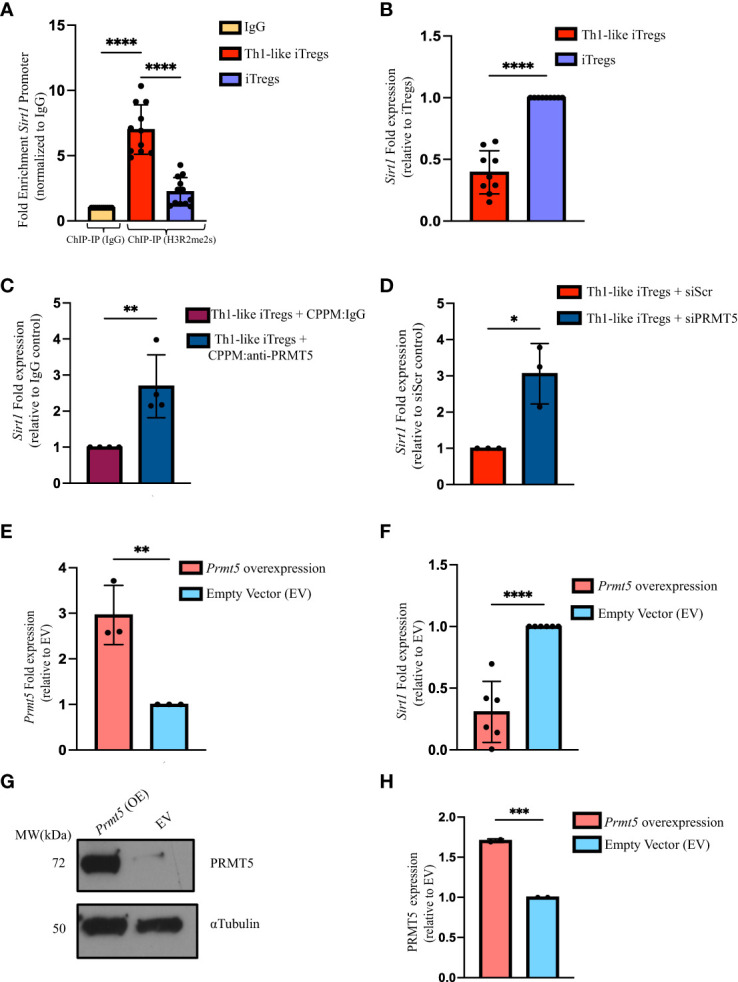
PRMT5 negatively regulates *Sirt1* transcription in Th1-like iTregs. **(A)** We verified the ChIP sequencing results using ChIP qPCR. We used anti-H3R2me2s to immunoprecipitate DNA bound to H3R2me2s from Th1-like iTregs (n=11) and iTregs (n=12), then amplified the bound DNA using primers specific for the *Sirt1* promoter region. We expressed H3R2me2s enrichment normalized to rabbit IgG (n=11). **(B)** We quantified *Sirt1* expression by qRT-PCR in Th1-like iTregs (n=9) and iTregs (n=9). We measured *Sirt1* expression in iTregs in which PRMT5 was knocked down using cell-penetrating peptide mimics to deliver **(C)** IgG (CPPM : IgG; control; n=4) or anti-PRMT5 (CPPM:anti-PRMT5; n=4) or siRNA approaches to deliver **(D)** scrambled siRNA (siScr; n=3) or PRMT5 siRNA (siPRMT5; n=3). **(E)** We overexpressed *Prmt5* (n=3) and measured **(F)** *Sirt1* (n=6), and **(G, H)** PRMT5 expression (n=2) in EV- or *Prmt5*-transduced samples. Relative gene expression is represented as fold expression and is determined using the 2-ΔΔCT method. Data are represented as fold expression of the gene, normalized to the housekeeping gene *ActB* (β-actin) and expressed relative to the controls in the respective experiments. Data are the mean ± SD and are representative of at least 2 experiments. Unpaired, two-tailed student’s *t* test; One-way ANOVA. *p < 0.05; ***p < 0.001 ****p < 0.0001.

To better define the mechanisms by which PRMT5 regulates Th1-like iTreg functions, and to distinguish its activity in Th1-like iTregs, compared to iTregs in general, we utilized ChIP sequencing to reveal target genes specific to Th1-like iTregs that may be regulated by H3R2me2s, as a measure of PRMT5 activity. Th1 cells also express H3R2me2s (**Supplementary Figure 4A**); therefore, we were interested in identifying genes whose promoters associated strongly with H3R2me2s and that were unique to Th1-like iTregs. We immunoprecipitated chromatin bound to H3R2me2s, then sequenced the bound DNA bound. **Figures 3A, B** represent the genome-wide binding patterns (peak distribution) for H3R2me2s in Th1-like iTregs and iTregs, respectively. Of interest, we noted that in Th1-like iTregs, H3R2me2s is enriched at the transcription start sites (TSS) in the promoter region of genes, compared to iTregs and Th1 cells, which show distinctly different binding patterns (**Figure 3C**; **Supplementary Figure 4B**). We also characterized the peak calling on the whole genome (**Supplementary Figure 4C**). We utilized M-A norm analysis to differentiate target genes that were uniquely found in Th1-like iTregs compared to iTregs (**Figure 3D**) or Th1 cells (**Supplementary Figure 5A**). Briefly, M-A norm analysis highlights genes with differences in the value of normalized read densities, as represented by the scaling relationship of the ChIP seq signals between two samples (36). Using this method, we identified the *Sirt1* promoter as one of the targets bound by H3R2me2s. Based on the normalized read density for *Sirt1* in Th1-like iTregs vs iTregs (5.33261 vs 0.84386, respectively), we found that significantly more H3R2me2s associated with the *Sirt1* promoter in Th1-like iTregs compared to iTregs (*p* value of 0.046875; data in raw file). To further verify this finding, we utilized integrative genome viewer (IGV) analysis to visualize the ChIP sequencing peak at the *Sirt1* promoter region. Compared to iTregs, the *Sirt1* promoter has a peak showing enriched association with H3R2me2s in Th1-like iTregs (**Figure 3E**; **Supplementary Figure 5B**). Finally, we used ChIP qPCR to verify H3R2me2s occupancy at the *Sirt1* promoter region, confirming that the *Sirt1* promoter region is enriched for H3R2me2s in Th1-like iTregs compared to the IgG control or to iTregs (**Figure 4A**).

### 3.4 PRMT5 negatively regulates *Sirt1* transcription in Th1-like iTregs

H3R2me2s occupancy at the *Sirt1* promoter provides strong evidence that *Sirt1* transcription is regulated by PRMT5; however, data in the literature indicate symmetric di-methylation can either enhance or inhibit gene transcription (37–39). To determine how H3R2me2s occupancy impacts *Sirt1* expression, we designed primers and used qPCR to assess *Sirt1* levels (**Supplementary Figure 5C**). We found that *Sirt1* mRNA expression is lower in Th1-like iTregs, compared to iTregs (**Figure 4B**). Sirt1 protein was also expressed at lower levels in Th1-like iTregs, compared to its expression in iTregs (**Supplementary Figures 5D, E**). These results indicated PRMT5 was likely acting to negatively regulate *Sirt1* in Th1-like iTregs. We again knocked-down PRMT5 using CPPM:anti-PRMT5 or siPRMT5 delivery, and observed *Sirt1* mRNA expression increased in Th1-like iTregs (**Figures 4C, D**, respectively). Finally, overexpressing *Prmt5* in Th1-like iTregs, reduced *Sirt1* transcripts (**Figures 4E, F**, respectively), in the presence of abundant PRMT5 (**Figures 4G, H**). Collectively, these data provide further evidence that the increased suppressive capacity of Th1-like iTregs is due to PRMT5 negatively regulating *Sirt1* transcription.

### 3.5 Th1-like iTregs ameliorate disease in a mouse model of aplastic anemia

**Figure 5:**
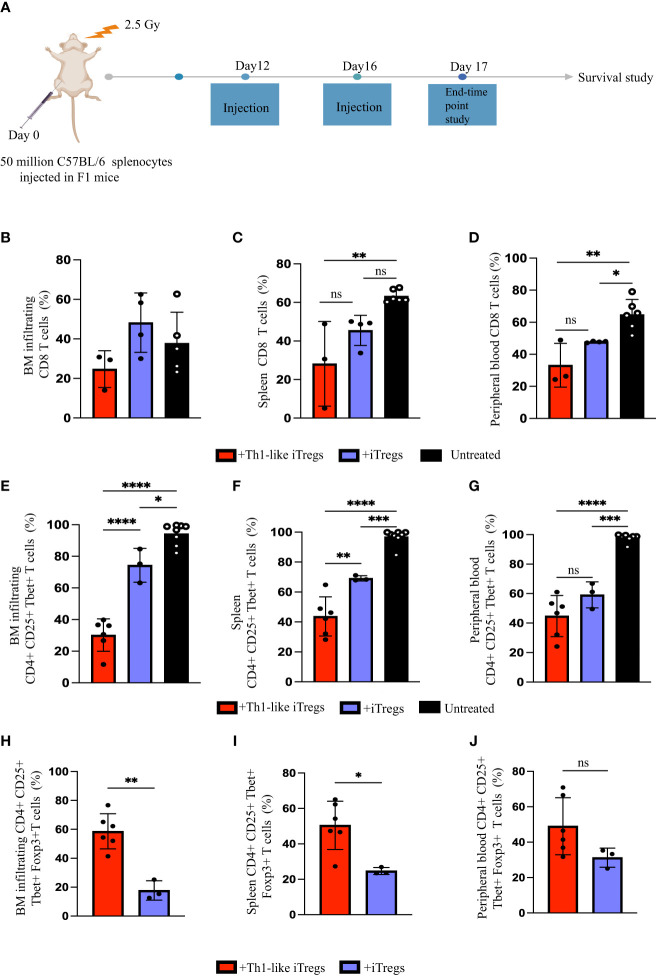
Th1-like iTregs ameliorate disease in a mouse model of Aplastic Anemia. **(A)** Experimental setup to induce disease and treat AA mice. C57BL/6 x Balb/c F1 mice were irradiated with 2.5 Gy, then injected intraperitoneally with 5x10^7^ C57BL/6 splenocytes to induce AA. On days 12 and 16 post-induction, mice were left untreated or treated with 2.5 x 10^6^ Th1-like iTregs or iTregs (injection). On day 17, some mice from each treatment group were humanely euthanized to further analyze BM, spleens, and peripheral blood. We used flow cytometry to determine the percentages of CD8+ cells in the **(B)** BM, **(C)** spleens, and **(D)** peripheral blood of AA mice left untreated (n=5) or treated on days 12 and 16 post-induction with Th1-like iTregs (n=3) or iTregs (n=4); the percentages of CD4+ CD25+ Tbet+ cells in the **(E)** BM, **(F)** spleen, and **(G)** peripheral blood in AA mice left untreated (n=7) or treated with Th1-like iTregs (n=6) or iTregs (n=3); and the percentages of CD4+ CD25+ Tbet+ Foxp3+ cells in the **(H)** BM, **(I)** spleen, and **(J)** peripheral blood in AA mice treated with Th1-like iTregs (n=6), or iTregs (n=3). Data are the mean ± SD and are representative of 3 independent experiments. Unpaired, two-tailed student’s *t* test. **p < 0.01. One-way ANOVA. *p < 0.05; **p < 0.01; ***p < 0.001; ****p < 0.0001; ns = no statistical difference.

**Figure 6:**
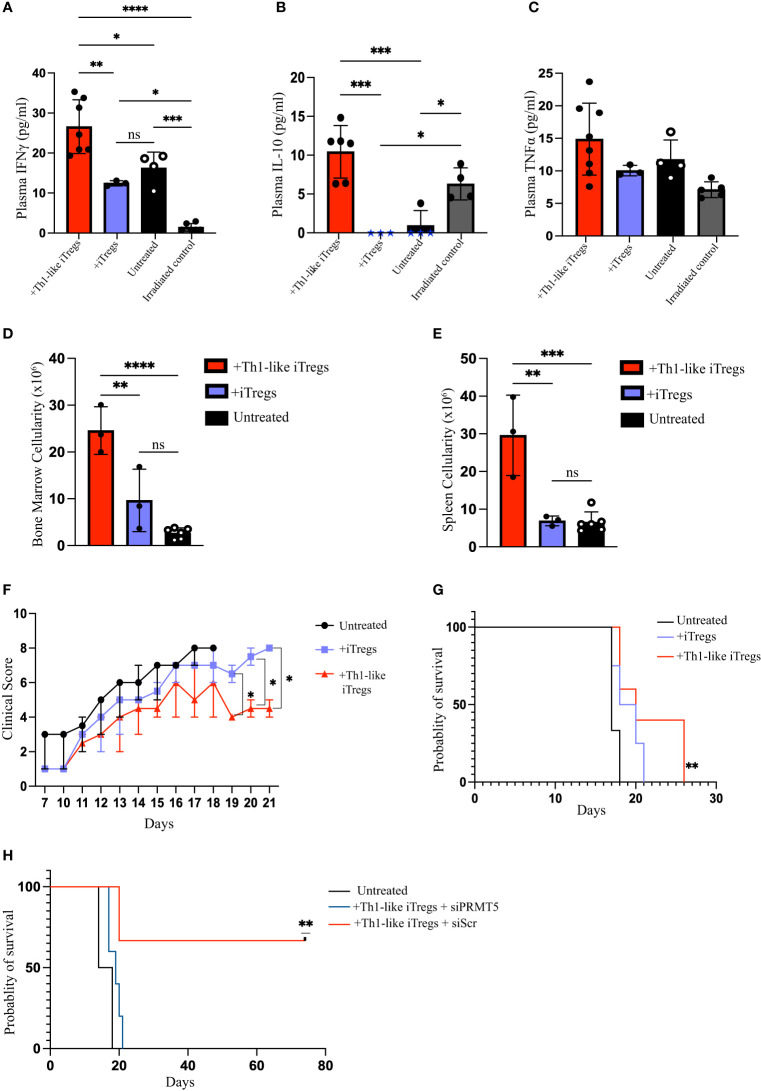
Administering Th1-like iTregs improve bone marrow cellularity and survival in mouse model of Aplastic Anemia. On day 17 post-disease induction, mice from each treatment group were humanely euthanized and BM, spleens, and peripheral blood were collected for analysis. We quantified circulating levels of IFNγ, IL-10, and TNFα for mice treated with Th1-like iTregs (n=6), iTregs (n=3), left untreated (n=4), and for irradiation controls (n=4). We used a bead-based flow cytometric assay to determine circulating levels of **(A)** IFNγ (pg/ml), **(B)** IL-10 (pg/ml), and **(C)** TNFα (pg/ml). We used Trypan Blue exclusion to determine cellularity in the **(D)** BM, and **(E)** spleens. For survival studies mice were scored daily beginning on day 10 post-disease induction and we monitored **(F)** clinical scores and **(G)** survival of AA mice left untreated (n=6) or treated on days 12 and 16 post-induction with Th1-like iTregs (n=4) or iTregs (n=4). To verify the contribution of PRMT5 to Th1-like iTreg suppression we monitored **(H)** the survival of AA mice left untreated (n=2) or treated on days 12 and 16 post-induction with Th1-like iTregs + siScr (n=3) or Th1-like iTregs + siPRMT5 (n=5). Data are the mean ± SD and are representative of 2 independent experiments. Kaplan-Meier analysis. **p < 0.01, One-way ANOVA. Two-way ANOVA. *p < 0.05; **p < 0.01; ***p < 0.001; ****p < 0.0001; ns = no statistical difference.

We previously demonstrated that aberrant responses mediated by Th1 cells play a significant role in the pathogenesis of AA in our mouse model (26). The *in vitro* study conducted by *Venigalla et al.*, showed that IFNγ-producing iTregs can arise from activated effector T cells and can downregulate Th1-mediated immune responses (40). Therefore, we hypothesized that Th1-like iTregs might ameliorate disease severity in our high-fidelity mouse model of immune mediated BMF. We induced AA by injecting 5x10^7^ million C57BL/6 splenocytes into the F1 offspring of C57BL/6 x BALB/c mice and followed disease severity, beginning 10 days post-induction. We injected 2.5 x 10^6^ therapeutic Th1-like iTregs or iTregs on days 12 and day 16 post-induction, when disease was well-established (**Figure 5A**), then assessed the cellular distribution in AA mice. Cytotoxic CD8 T cells, and Th1 cells, together with impaired Treg function, collectively contribute to AA pathogenesis (41). The percentages CD8 T cells in the BM were similar across treatment cohorts (**Figure 5B**); however, the absolute number of BM-infiltrating CD8 T cells were significantly lower in AA mice treated with Th1-like iTregs or iTregs, compared to untreated mice (**Supplementary Figures 6A–C**). The percentages of CD8 T cells in the spleen and peripheral blood showed this same trend, indicating Th1-like iTreg- or iTreg-treatment can reduce cytotoxic CD8 T cells (**Figures 5C, D**; **Supplementary Figures 6B, C**), and supports the notion that, in general, iTregs can reduce cytotoxic CD8 T cell populations in AA mice. Th1 cells are implicated in driving AA development and disease progression (26, 27), so we analyzed the pathogenic CD4+CD25+Tbet+ T cell population that did not express Foxp3. This approach allowed us to specifically focus on the pathogenic Th1 cells while excluding the injected Th1-like iTregs, which typically express high levels of the transcription factor Foxp3. The percentages of CD4+, CD25+, and Tbet+ T cells in the BM, spleen, and peripheral blood of mice treated with Th1-like iTregs were all significantly reduced, compared to mice left untreated or treated with iTregs (**Figures 5E–G**), as were the absolute numbers (**Supplementary Figures 6D–F**). There were also increased percentages, as well as absolute numbers, of Th1-like iTregs in BM, spleen, and peripheral blood of Th1-like-treated mice demonstrating Th1-like iTregs were successfully migrating to relevant immune compartments to attenuate disease (**Figures 5H–J**; **Supplementary Figures 6G–I**). This migration, especially to the BM, is crucial for Th1-like iTregs to modulate immune responses and ameliorate symptoms in AA mice. To determine whether Th1-like iTreg treatment affected the balance of pro- and anti-inflammatory cytokines, such as IFNγ, IL-10, and TNFα, we analyzed circulating cytokine levels in the plasma of untreated and treated mice on day 17 after disease induction. IFNγ and IL-10 were significantly higher in mice treated with Th1-like iTregs compared to mice treated with iTregs, left untreated, or to irradiation controls (**Figures 6A, B**). However, we did not observe significant changes in TNFα levels between treatment cohorts (**Figure 6C**). As an anti-inflammatory cytokine, IL-10 regulates immune homeostasis. Conversely, although increased IFNγ can lead to inflammation *in vivo*, IFNγ-producing, highly suppressive iTregs have been previously described, including by our lab (34). The fact that circulating IL-10 levels were elevated only in mice treated with Th1-like iTregs supports the idea that the balance between pro- and anti-inflammatory cytokine signaling likely contributes to the overall improved clinical outcome for AA mice treated with Th1-like iTregs. The precise cytokine signals that promote, or protect against, bone marrow failure in our model of AA, especially in the context of Th1-like iTreg treatment, are undoubtedly complex and warrant further in-depth investigation.

### 3.6 Administering Th1-like iTregs improve bone marrow cellularity and survival in a mouse model of aplastic anemia

Hematopoietic cells in the BM are destroyed during AA disease progression, resulting in BM hypocellularity that is fatal if left untreated. However, mice treated with Th1-like iTregs showed greater cellularity in their BM and spleens, compared to untreated or iTreg-treated AA mice (**Figures 6D, E**). Consistent with this finding, mice treated with Th1-like iTregs displayed less severe clinical symptoms, based on a standard scoring rubric, compared to mice left untreated or to mice treated with iTregs (**Figure 6F**). We also evaluated the survival benefit of treating mice with Th1-like iTregs. Untreated mice succumbed to lethal bone marrow failure, on average, by day 18 post-disease induction. Mice treated with iTregs survived slightly longer, the difference in survival between untreated and iTreg-treated cohorts did not reach statistical significance. In stark contrast, mice treated with Th1-like iTregs survived significantly longer compared to untreated mice, or to mice treated with iTregs (**Figure 6G**), indicating Th1-like iTreg treatment provides a survival benefit, even when given during the active stages of AA disease progression. Finally, to confirm that PRMT5 mediates the therapeutic activity of Th1-like iTreg-treatment, we knocked down *Prmt5* in Th1-like iTregs using siRNA approaches, treated AA mice with these cells on days 12 and 16, and followed their survival. We found that downregulating PRMT5 in Th1-like iTregs abrogated their therapeutic potential and reduced the survival of AA mice to that of the untreated cohort, while Th1-like iTregs transduced with scrambled siRNA still conveyed a significant survival benefit (**Figure 6H**). Altogether, on the strength of our *in vitro* and *in vivo* data, our study demonstrates that PRMT5 is critically important for Th1-like iTreg therapeutic capacity and administering Th1-like iTregs under clinically relevant conditions can attenuate disease severity and improve survival in AA mice.

## 4 Discussion

Tregs are not considered to be irreversibly committed to their cell lineage. Rather, studies show they retain some degree of plasticity. This functional cell plasticity is an intrinsic property of most immune cells which helps them to adopt different phenotypes and functions in response to changing environments (42–44). In this study, we focused on iTreg plasticity and Treg-specific epigenetic patterns and demonstrated that Th1-like iTregs acquire distinct epigenetic patterns after exposure to the Th1-promoting cytokine, IL-12. The balance between iTregs differentiating towards suppressive, Th1-like iTregs with a functional phenotype, or a pathogenic/dysfunctional state depends on the level of Th1 cytokines, IFNγ or IL-12, in the microenvironment (44). We used a 7-day differentiation protocol to show that adding IL-12 into the iTreg differentiation media on day 3 of polarization generated suppressive Th1-like iTregs which expressed increased levels of Foxp3, Tbet, and IFNγ. We also found that exposing iTregs to IL-12 during the initial phase of differentiation increased their capacity to suppress anti-CD3 plus anti-CD28-activated responders, as well as alloactivated responders.

The molecular pathways that mediate suppressive activity in Th1-like iTregs is an area that requires further investigation, especially for developing targeted therapeutic strategies aimed at enhancing their regulatory capacity. We observed upregulated PRMT5 enzymatic activity (H3R2me2s) in activated T cells (data not shown) and Th1 cells, but not in iTregs. Rather, we demonstrated that IL-12 treatment on day 3 modulated multiple components of iTreg differentiation, including increased PRMT5 enzymatic activity, as measured by H3R2me2s expression. We extended these findings using reciprocal experiments and showed that using siRNA, or cell-penetrating antibodies to inhibit PRMT5, attenuated H3R2me2s expression in Th1-like iTregs and abrogated their superior suppressive capacity. Using ChIP-sequencing, we demonstrated that H3R2me2s binds to the TSS on the *Sirt1* promoter to modulate its transcription in a way that is unique to Th1-like iTregs, and not observed in iTregs or Th1 cells, revealing an as-yet-undescribed means by which H3R2me2s epigenetically regulates genes in Th1-like iTregs.

Foxp3 is crucial for Treg development, function, and maintenance, and deleting Foxp3 in iTregs abrogates their suppressive activity (45). As a result, Foxp3 expression in Treg is tightly regulated. *Foxp3* transcription is modulated at an epigenetic level, and protein expression can be further controlled through post-translational modifications (46). Sirt1 destabilizes iTregs by deacetylating Foxp3, facilitating its proteasomal degradation. Several studies have shown that downregulating *Sirt1*, or Treg-specific *Sirt1* deletion, generates stable and highly suppressive iTregs (22–24). We found that H3R2me2s occupies the *Sirt1* promoter region and downregulates its transcription. Reducing PRMT5 expression increased *Sirt1* transcription in Th1-like iTregs, providing strong evidence PRMT5 regulates *Sirt1* transcription through epigenetic modifications in Th1-like iTregs. Our data indicated that PRMT5 can inhibit *Sirt1* transcription in Th1-like iTregs, to enhance Foxp3 stability and increase their suppressive capabilities. Whether PRMT5 enhances Th1-like iTreg function solely by negatively regulating *Sirt1*, or if it contributes to Th1-like iTreg stability through multiple mechanisms such as modulating autophagy, metabolism, or other transcription factors constitutes an area of ongoing investigation by our lab.

In certain autoimmune diseases like type 1 diabetes, multiple sclerosis, autoimmune hepatitis, and Sjogren syndrome, there is an increased frequency of IFNγ+Foxp3+ thymic Treg cells in the peripheral blood. These cells often exhibit reduced suppressive capacities compared to Treg cells from healthy individuals (47). While some reports indicate these Th1-like iTregs may contribute to disease progression by promoting inflammation or loss of immune tolerance (46), other studies propose that they might represent a compensatory response aimed at controlling excessive immune activation (31, 48). The precise mechanisms underlying the functional changes observed in Th1-like iTregs during autoimmunity are still unclear, and it is likely that the inflammatory microenvironment within affected tissues influences Th1-like iTreg differentiation and function. Additionally, genetic factors and epigenetic modifications may also contribute to the altered behavior of these cells.

In a recent study, Gocher-Demske et al., showed that Th1-like iTregs could detect inflammatory signals, exert regulatory control over immune responses, prevent prolonged immunoinflammatory reactions, and influence the quality and quantity of memory T-cell responses during acute and chronic viral infections (31). In our *in vivo* study using a Th1-mediated mouse model of AA, therapeutically administering Th1-like iTregs improved clinical scores and extended survival. Further, in-depth analysis shows that in Th1-like iTreg-treated mice, there were reduced percentages of pathogenic Th1 cells, as well as CD8 T cells, in their BM, spleen, and peripheral blood. These were accompanied by elevated levels of circulating cytokines, including IFNγ and IL-10. Collectively, these results support the notion that Th1-like iTregs can be protective in Th1-dominant immune disorders.

As such, our data point to the potential for using Th1-like iTregs as cell based immunosuppressive therapy for AA, although care must be taken when extrapolating data from animal models. In a clinical setting, one could envision the patient’s own peripheral blood mononuclear cells (PBMCs) being utilized to generate suppressive Th1-like iTregs. However, further detailed studies are needed to determine the clinical relevance of administering Th1-like iTregs, their stability *in vivo*, and even the feasibility of patients sourcing sufficient PBMCs to generate Th1-like iTregs, since pancytopenia is a common symptom of AA. Further, it remains unknown whether Th1-like iTregs transform into “ex-Foxp3” cells over time, which could have negative consequences for patient use. At the same time, how Th1-like iTregs behave or function in the presence of other T helper cell subsets, such as Th2 or Th17 cells, can be complex and may vary depending on the specific disease and microenvironment. Th17 cells have been identified in patients with AA, and correlated with disease progression (49), so understanding how Th1-like iTregs may affect cross-regulation between T helper subsets certainly warrants further investigation. The outcomes of such interactions can be influenced by various factors, including the cytokine milieu, tissue microenvironment, genetic factors, and epigenetic modifications, all of which can shape the plasticity and functional properties of effector and regulatory T cells within a specific disease context. Therefore, it will be important to consider the dynamic nature of immune responses and to gain a more thorough understanding of how Th1-like iTregs interact with other T helper cell subsets to influence disease severity and progression in AA as well as other Th 1 cell-mediated diseases.

PRMT5 is necessary for T-cell survival, cytokine production, homeostasis, and iTreg suppressive capacity (14, 15). In this study, we have demonstrated that this epigenetic modulator contributes to the potent suppressive activity of Th1-like iTregs by negatively regulating *Sirt1*. Increasing our understanding of iTreg differentiation and plasticity will improve Treg-specific therapeutic options for patients with autoimmune disorders, including patients with AA. By addressing gaps in our knowledge, we can gain a deeper understanding of Th1-like iTreg biology and how PRMT5 contributes to their stable phenotype and potential as a suppressive cell-based therapy.

## Data availability statement

The data presented in the study are deposited in the Gene Expression Omnibus (GEO) repository, accession number GSE243686.

## Ethics statement

The animal study was approved by Institutional Animal Care and Use Committee (IACUC), University of Massachusetts Amherst, Amherst, MA 01003, USA. The study was conducted in accordance with the local legislation and institutional requirements.

## Author contributions

NJ: Conceptualization, Writing – review & editing, Investigation, Methodology, Writing – original draft. SS: Methodology, Writing – review & editing. GT: Writing – review & editing, Funding acquisition, Project administration. LM: Funding acquisition, Project administration, Conceptualization, Supervision, Writing – review & editing.

## Supplementary material

Supplementary Figure 1 Generating Th1-like iTregs. CD4 T cells were isolated from spleens of C57BL/6 mice, resuspended into iTreg differentiation media, and polarized for 7 days. Samples were left untreated or treated with IL-12 (10ng/mL) on day 3 and/or day 5 of polarization. On day 7, cells were harvested and analyzed by flow cytometry for the expression of **(A)** CD25 (n=5), **(B)** Foxp3 (n=3), **(C)** Tbet (n=3), **(D)** IFNγ (n=3), **(E)** the percentages of Foxp3^+^Tbet^+^IFNγ^+^ cells (n=3), **(F)** CTLA-4 (n=2), **(G)** PD-1 (n=2). MFI = Mean Fluorescence Intensity. We collected supernatants from suppression assays and used ELISA to quantify secreted **(H)** IL-10 (pg/ml; n=3), and **(I)** IFNγ (pg/ml; n=3). **(H)** We determined the percent suppression of C57BL/6 splenocytes (responders) by Th1-like iTregs (n=3) and iTregs (n=3) treated with anti-IFNγ (1μg/ml) during their polarization process. Data are the mean ± SD and are representative of at least 2 experiments. One-way ANOVA. **p* < 0.05; ***p* < 0.01; ****p* < 0.001; ns = no statistical difference.

Supplementary Figure 2 siRNA delivery into iTregs and Th1-like iTregs.CD4T cells were pre-activated for 24 hours before transfection with siRNA, then cells were differentiated for 7 days using the standard differentiation protocol. **(A)** We visualized and quantified temporal PRMT5 expression in iTregs treated with different concentrations of PRMT5 siRNA to titrate siRNA concentrations for *Prmt5* knockdown (n=1). Th1-like iTregs or iTregs transfected with scrambled (siScr) or PRMT5 siRNA (siPRMT5) and cultured without or with IL-12 on day 3 of polarization (n=3). On day 7, cells were harvested and analyzed by flow cytometry for the expression of **(B)** CD25, **(C)** Foxp3, **(D)** Tbet, and **(E)** IFNγ. We stimulated responder cells (Tresp) with soluble anti-CD3ϵ plus anti-CD28, cross-linked antibody-bound receptors with hamster IgG, then determined the percent at which their proliferation was suppressed by **(F)** iTregs treated with scrambled (siScr; n=5) or PRMT5 siRNA (siPRMT5; n=5). Data are the mean ± SD and are representative of at least 3 experiments. One-way ANOVA. **p* < 0.05; ***p* < 0.01; ****p* < 0.001; ns=no statistical difference; two-way ANOVA. **p* < 0.05; ****p* < 0.001; *****p* < 0.0001.

Supplementary Figure 3
*In vitro* CPPM-antibody complex delivery into iTregs andTh1-like iTregs. **(A)** Experimental setup for *in vitro* Th1-like iTreg differentiation protocol following cell-penetrating IgG (CPPM : IgG) or cell-penetrating anti-PRMT5 (CPPM:anti-PRMT5) delivery. **(B)** Immunoblot of PRMT5 following CPPM : IgG or CPPM:anti-PRMT5 delivery to Th1-like iTregs. **(C)** Quantified PRMT5 expression from CPPM:anti-PRMT5-treated samples, normalized to α-tubulin and expressed relative to PRMT5 levels in cells treated with CPPM : IgG. Th1-like iTregs and iTregs were treated with CPPM : IgG or CPPM:anti-PRMT5 and cultured without or with IL-12 on day 3 of polarization (n=4). On day 7, cells were harvested and analyzed by flow cytometry for the expression of **(D)** CD25, **(E)** Foxp3, **(F)** Tbet, and **(G)** IFNγ. We stimulated responder cells (Tresp) with soluble anti-CD3ϵ plus anti-CD28, cross-linked antibody-bound receptors with hamster IgG, then determined the percent at which their proliferation was suppressed by **(H)** Th1-like iTregs (Tsupp) treated with CPPM : IgG or CPPM:anti-PRMT5 (n=6) or **(I)** iTregs (Tsupp) treated with CPPM : IgG or CPPM:anti-PRMT5 (n=6). Band densities of immunoblots were quantified using ImageJ. Data are the mean ± SD and are representative of at least 3 experiments. Unpaired, two-tailed student’s *t* test; One-way ANOVA. **p* < 0.05; ****p* < 0.001; ***p* < 0.01; two-way ANOVA. **p* < 0.05; ****p* < 0.001; *****p* < 0.0001.

Supplementary Figure 4 Genome-wide profiling of H3R2me2s-1. **(A)** Representative immunoblot of PRMT5 and H3R2me2s expression in Th1 cells harvested on day 7 of polarization (n=3). **(B)** Heatmap and representative graph of H3R2me2s ChIP-sequencing of Th1-like iTregs (n=2), iTregs (n=1), and Th1 cells (n=1). **(C)** Crisscross representation of peak calling on chromosomes.

Supplementary Figure 5 Genome-wide profiling of H3R2me2s-2. **(A)** M-A norm analysis of genes H3R2me2s associates with in Th1-like iTregs (Th1-like iTregs unique; red), Th1 cells (Th1 unique; blue), and in both cell types (common genes; grey). **(B)** We used the integrative genomic viewer (IGV) to visualize peaks at the *Sirt1* promoter. **(C)** Schematic representation of *Sirt1* ChIP sequencing peak location on chromosome 10 and targeting primers used for ChIP qPCR. **(D, E)** Representative immunoblot and quantification of Sirt1 expressed in iTregs (n=2) and Th1-like iTregs (n=2). Band densities on the immunoblot were quantified using ImageJ.

Supplementary Figure 6 Th1-like iTregs as a cell-based therapy for AA. We used flow cytometry to analyze immune cell populations in the BM, spleens, and peripheral blood of AA mice. We calculated absolute numbers of CD8 T cells in the **(A)** BM, **(B)** spleens, and **(C)** peripheral blood from AA mice left untreated (n=6) or treated on days 12 and 16 post-induction with Th1-like iTregs (n=3) or iTregs (n=4). We determined absolute numbers of CD4+ CD25+ Tbet+ cells in the **(D)** BM, **(E)** spleens, and **(F)** peripheral blood from AA mice left untreated (n=5) or treated with Th1-like iTregs (n=5), or iTregs (n=3). We measured the absolute numbers of CD4+ CD25+ Tbet+ Foxp3+ cells in the **(G)** BM, **(H)** spleens, and **(I)** peripheral blood from AA mice treated with Th1-like iTregs (n=5), or iTregs (n=3). Data are the mean ± SD and are representative of 3 independent experiments. One-way ANOVA. **p* < 0.05; ***p* < 0.01; ****p* < 0.001; *****p* < 0.0001; ns=no statistical difference.

Supplementary Table 1 Primers used for Real-Time Quantitative PCR.

Supplementary Table 2 Primers used for ChIP qPCR.
