# Supplementary figures and images for: PRMT5 regulates epigenetic changes in suppressive Th1-like iTregs in response to IL-12 treatment
Source: Front Immunol. 2024 Jan 8;14:1292049. doi: 10.3389/fimmu.2023.1292049 (PMC10800960; doi:10.3389/fimmu.2023.1292049)

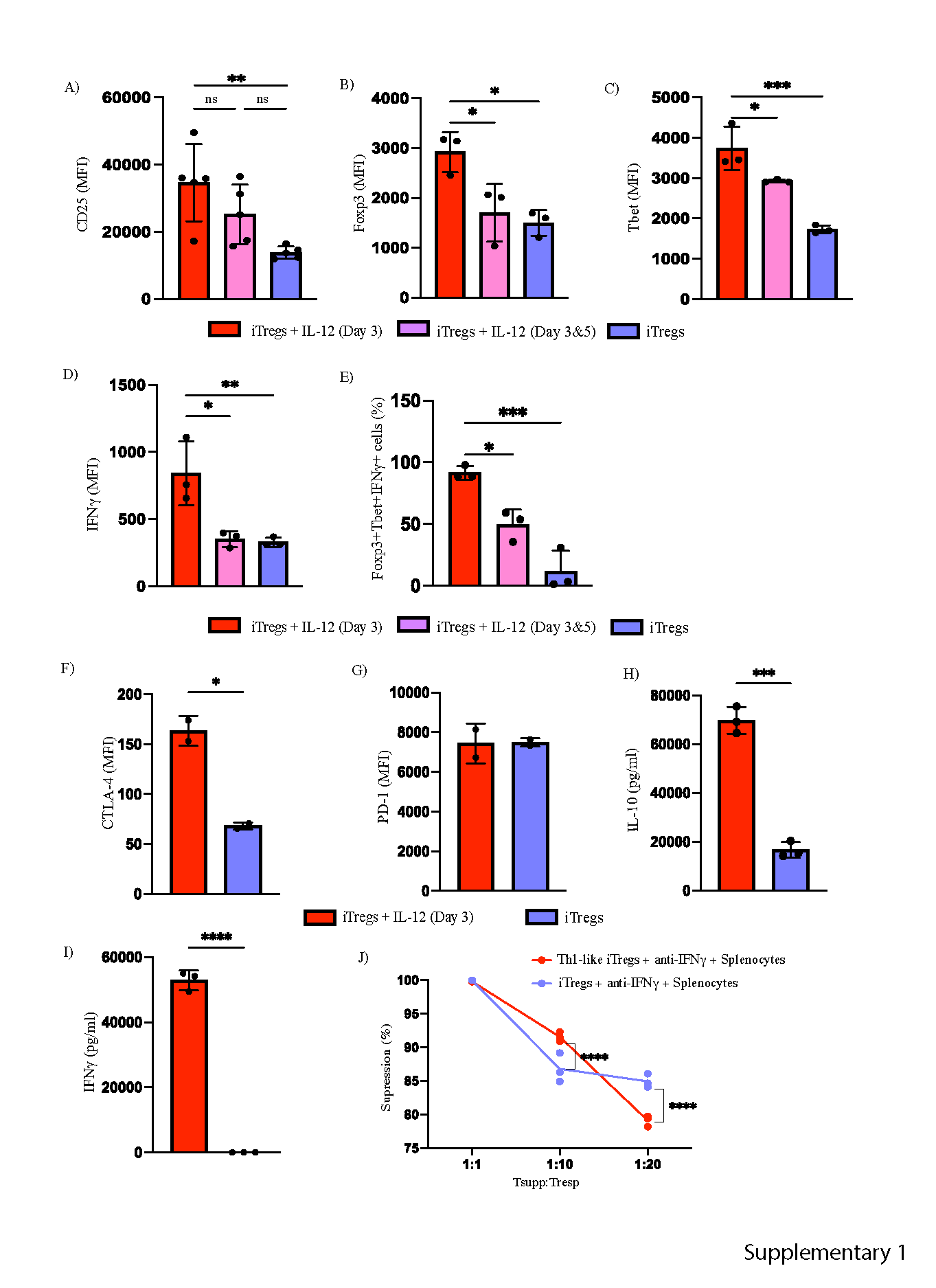

Supplement: Supplementary Figure 1 — Generating Th1-like iTregs. CD4 T cells were isolated from spleens of C57BL/6 mice, resuspended into iTreg differentiation media, and polarized for 7 days. Samples were left untreated or treated with IL-12 (10ng/mL) on day 3 and/or day 5 of polarization. On day 7, cells were harvested and analyzed by flow cytometry for the expression of (A) CD25 (n=5), (B) Foxp3 (n=3), (C) Tbet (n=3), (D) IFNγ (n=3), (E) the percentages of Foxp3+Tbet+IFNγ+ cells (n=3), (F) CTLA-4 (n=2), (G) PD-1 (n=2). MFI = Mean Fluorescence Intensity. We collected supernatants from suppression assays and used ELISA to quantify secreted (H) IL-10 (pg/ml; n=3), and (I) IFNγ (pg/ml; n=3). (H) We determined the percent suppression of C57BL/6 splenocytes (responders) by Th1-like iTregs (n=3) and iTregs (n=3) treated with anti-IFNγ (1μg/ml) during their polarization process. Data are the mean ± SD and are representative of at least 2 experiments. One-way ANOVA. *p < 0.05; **p < 0.01; ***p < 0.001; ns = no statistical difference. [file Image_1.tif]

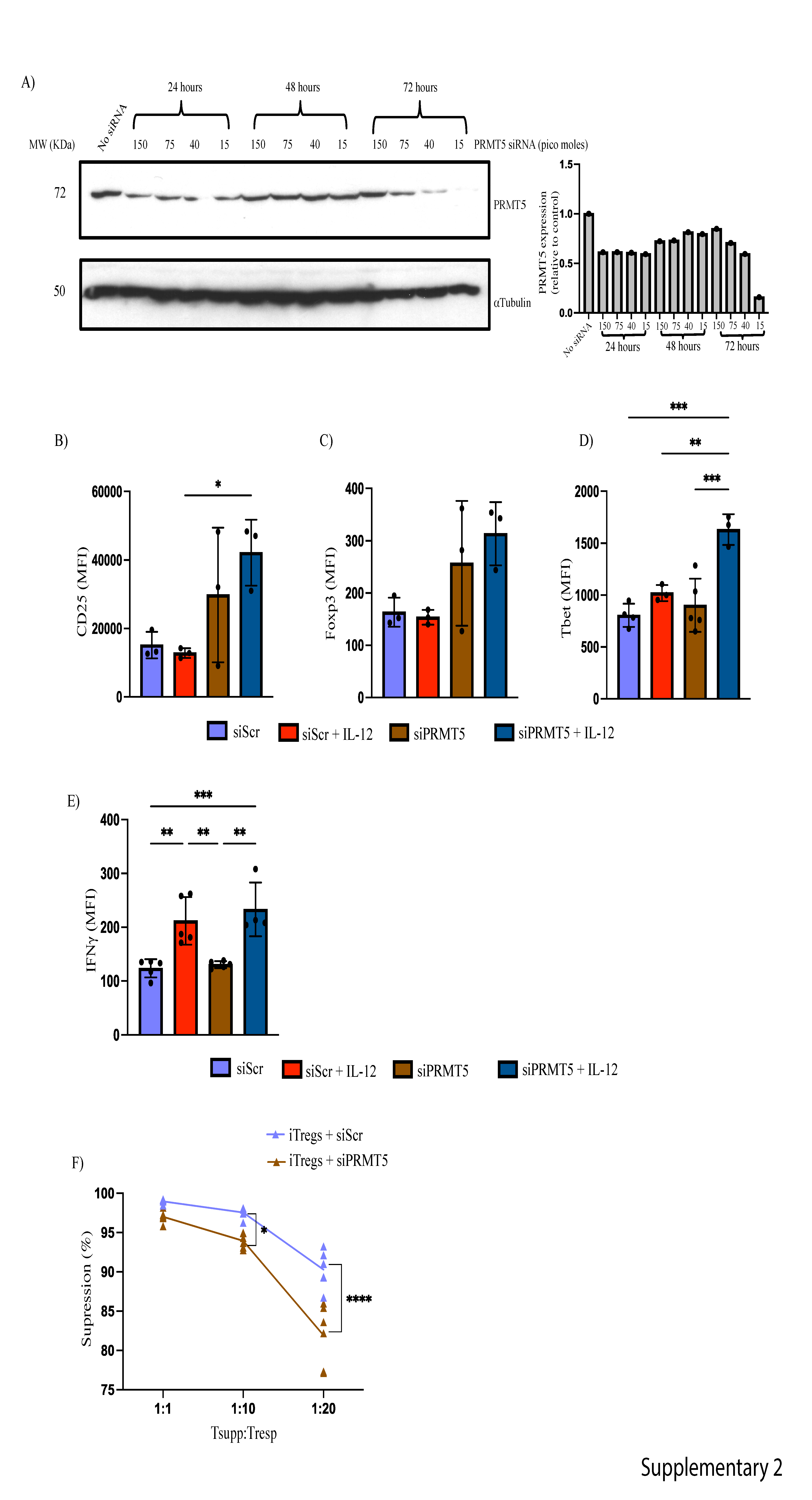

Supplement: Supplementary Figure 2 — siRNA delivery into iTregs and Th1-like iTregs.CD4T cells were pre-activated for 24 hours before transfection with siRNA, then cells were differentiated for 7 days using the standard differentiation protocol. (A) We visualized and quantified temporal PRMT5 expression in iTregs treated with different concentrations of PRMT5 siRNA to titrate siRNA concentrations for Prmt5 knockdown (n=1). Th1-like iTregs or iTregs transfected with scrambled (siScr) or PRMT5 siRNA (siPRMT5) and cultured without or with IL-12 on day 3 of polarization (n=3). On day 7, cells were harvested and analyzed by flow cytometry for the expression of (B) CD25, (C) Foxp3, (D) Tbet, and (E) IFNγ. We stimulated responder cells (Tresp) with soluble anti-CD3ϵ plus anti-CD28, cross-linked antibody-bound receptors with hamster IgG, then determined the percent at which their proliferation was suppressed by (F) iTregs treated with scrambled (siScr; n=5) or PRMT5 siRNA (siPRMT5; n=5). Data are the mean ± SD and are representative of at least 3 experiments. One-way ANOVA. *p < 0.05; **p < 0.01; ***p < 0.001; ns=no statistical difference; two-way ANOVA. *p < 0.05; ***p < 0.001; ****p < 0.0001. [file Image_2.tif]

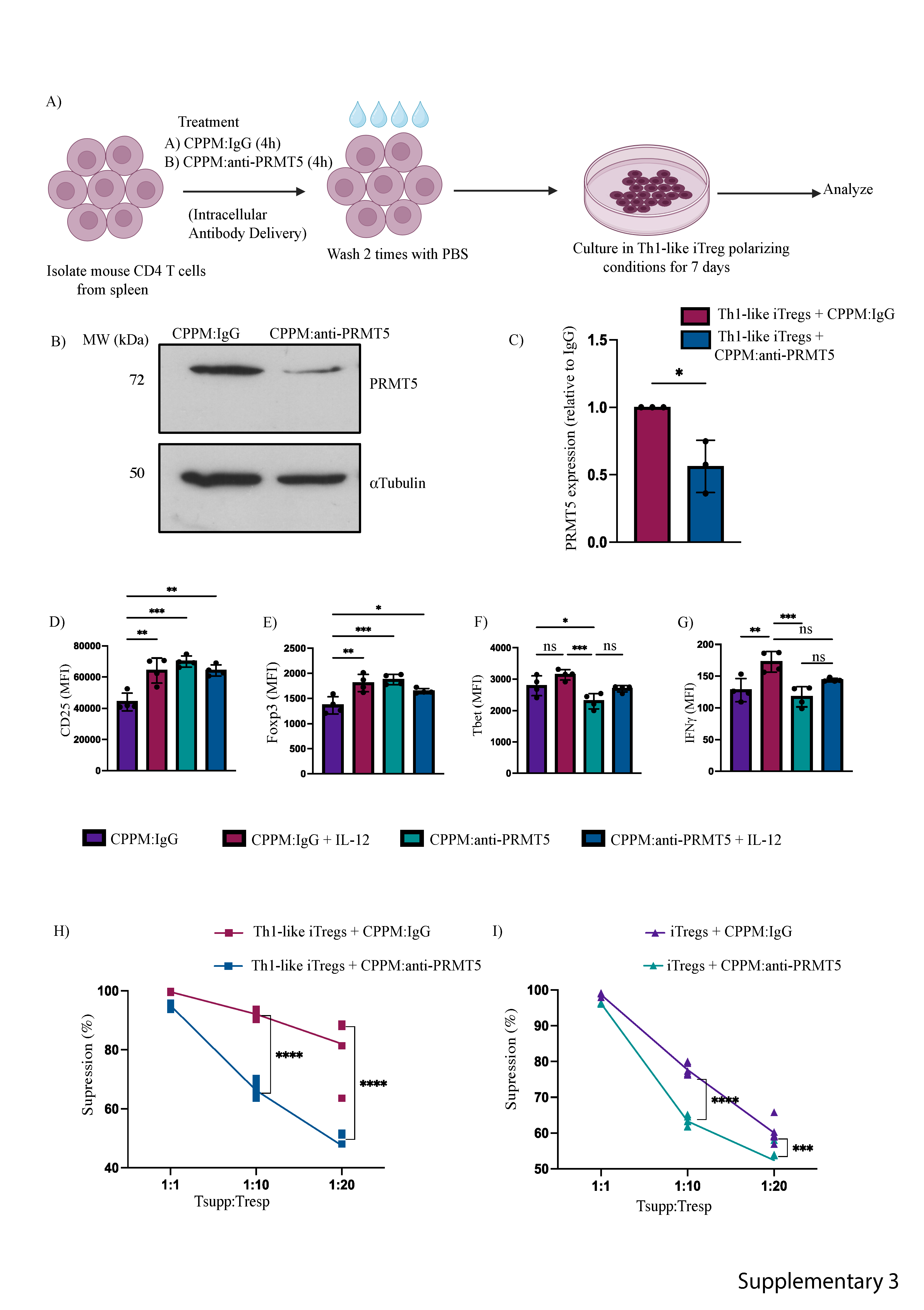

Supplement: Supplementary Figure 3 — In vitro CPPM-antibody complex delivery into iTregs andTh1-like iTregs. (A) Experimental setup for in vitro Th1-like iTreg differentiation protocol following cell-penetrating IgG (CPPM : IgG) or cell-penetrating anti-PRMT5 (CPPM:anti-PRMT5) delivery. (B) Immunoblot of PRMT5 following CPPM : IgG or CPPM:anti-PRMT5 delivery to Th1-like iTregs. (C) Quantified PRMT5 expression from CPPM:anti-PRMT5-treated samples, normalized to α-tubulin and expressed relative to PRMT5 levels in cells treated with CPPM : IgG. Th1-like iTregs and iTregs were treated with CPPM : IgG or CPPM:anti-PRMT5 and cultured without or with IL-12 on day 3 of polarization (n=4). On day 7, cells were harvested and analyzed by flow cytometry for the expression of (D) CD25, (E) Foxp3, (F) Tbet, and (G) IFNγ. We stimulated responder cells (Tresp) with soluble anti-CD3ϵ plus anti-CD28, cross-linked antibody-bound receptors with hamster IgG, then determined the percent at which their proliferation was suppressed by (H) Th1-like iTregs (Tsupp) treated with CPPM : IgG or CPPM:anti-PRMT5 (n=6) or (I) iTregs (Tsupp) treated with CPPM : IgG or CPPM:anti-PRMT5 (n=6). Band densities of immunoblots were quantified using ImageJ. Data are the mean ± SD and are representative of at least 3 experiments. Unpaired, two-tailed student’s t test; One-way ANOVA. *p < 0.05; ***p < 0.001; **p < 0.01; two-way ANOVA. *p < 0.05; ***p < 0.001; ****p < 0.0001. [file Image_3.tif]

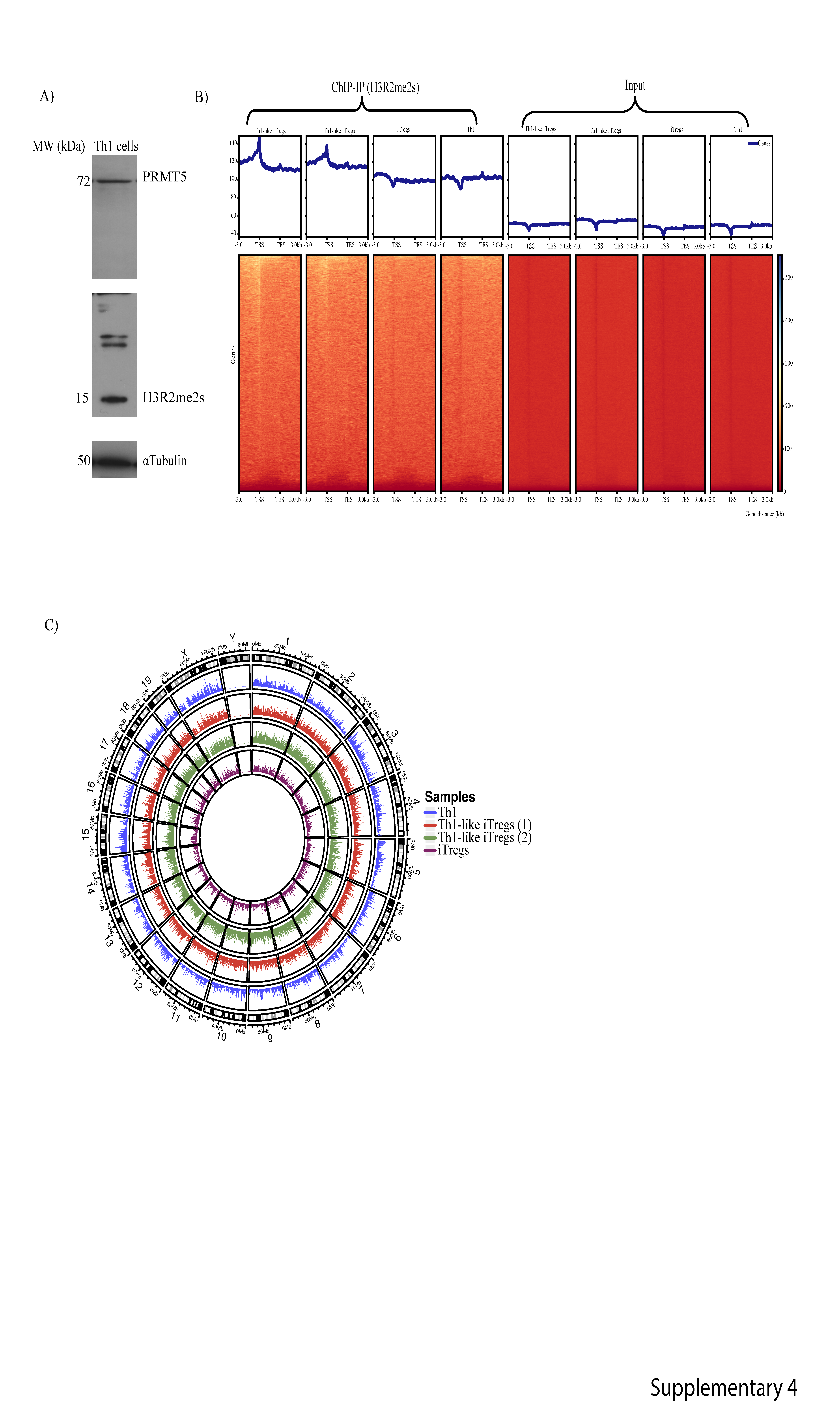

Supplement: Supplementary Figure 4 — Genome-wide profiling of H3R2me2s-1. (A) Representative immunoblot of PRMT5 and H3R2me2s expression in Th1 cells harvested on day 7 of polarization (n=3). (B) Heatmap and representative graph of H3R2me2s ChIP-sequencing of Th1-like iTregs (n=2), iTregs (n=1), and Th1 cells (n=1). (C) Crisscross representation of peak calling on chromosomes. [file Image_4.tif]

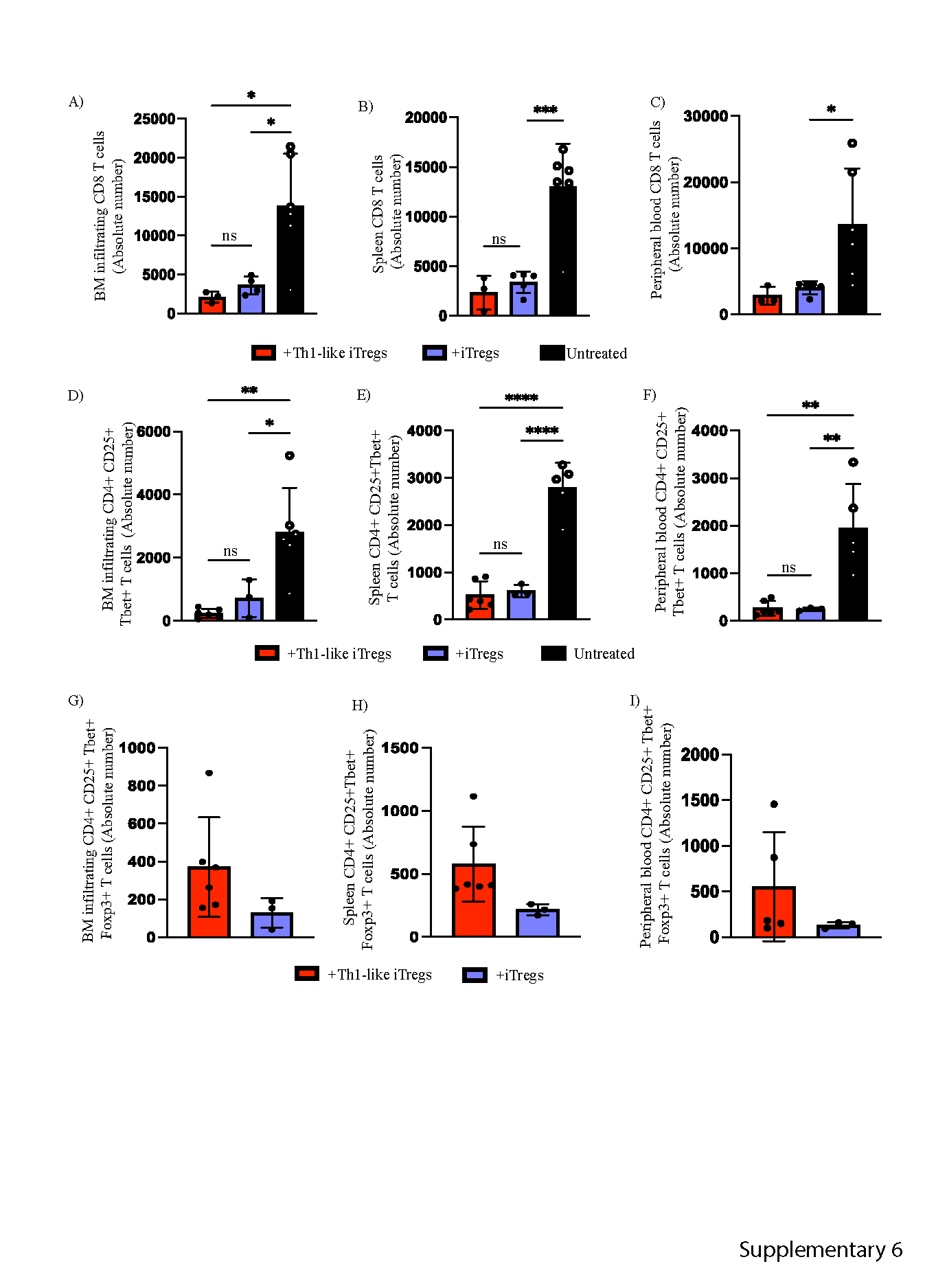

Supplement: Supplementary Figure 6 — Th1-like iTregs as a cell-based therapy for AA. We used flow cytometry to analyze immune cell populations in the BM, spleens, and peripheral blood of AA mice. We calculated absolute numbers of CD8 T cells in the (A) BM, (B) spleens, and (C) peripheral blood from AA mice left untreated (n=6) or treated on days 12 and 16 post-induction with Th1-like iTregs (n=3) or iTregs (n=4). We determined absolute numbers of CD4+ CD25+ Tbet+ cells in the (D) BM, (E) spleens, and (F) peripheral blood from AA mice left untreated (n=5) or treated with Th1-like iTregs (n=5), or iTregs (n=3). We measured the absolute numbers of CD4+ CD25+ Tbet+ Foxp3+ cells in the (G) BM, (H) spleens, and (I) peripheral blood from AA mice treated with Th1-like iTregs (n=5), or iTregs (n=3). Data are the mean ± SD and are representative of 3 independent experiments. One-way ANOVA. *p < 0.05; **p < 0.01; ***p < 0.001; ****p < 0.0001; ns=no statistical difference. [file Image_6.tif]
