# Supplementary material for: PRMT5 regulates epigenetic changes in suppressive Th1-like iTregs in response to IL-12 treatment
Source: Front Immunol. 2024 Jan 8;14:1292049. doi: 10.3389/fimmu.2023.1292049 (PMC10800960; doi:10.3389/fimmu.2023.1292049)
Supplement: Supplementary Table 1 — Primers used for Real-Time Quantitative PCR. [file Table_1.docx]

**Supplementary Table 1.**

**Primers used for Real-Time Quantitative PCR**

| Gene | Forward | Reverse |
| --- | --- | --- |
| *Sirt1* | 5’-TAGTTCTGACTGGAGCTGG-3’ | 5’-CTGGAACTGTCCGGGAT-3’ |
| *Actb* | 5’-CGGTTCCGATGCCCTGAGGCTCTT-3’ | 5’-CGTCACACTTCATGATGGAATTGA-3’ |
